# Toxicant contents of oral nicotine pouches: comparative quantitative analysis with Swedish snus and cigarette smoke

**DOI:** 10.1186/s13065-026-01895-x

**Published:** 2026-08-14

**Authors:** Simone Hadley, Belinda Zonnestein, Jana Jeffery, Yen Nguyen, Kevin McAdam, Helena Digard

**Affiliations:** 1 BAT (Investments) Limited, Regents Park Road, Southampton, SO15 8TL UK; 2 McAdam Scientific Ltd., River Bank House, 65A Bishopstoke Road, Eastleigh, Hampshire SO50 6BF UK

**Keywords:** Oral nicotine pouches, Swedish snus, Tobacco harm reduction, Chemical analysis

## Abstract

**Background and aims:**

Swedish snus is a smokeless tobacco product (STP) associated with potentially reduced health risks compared to conventional cigarettes, due in large part to the absence of high toxicant levels generated by combustion. In 2019 and renewed in 2024, the US Food and Drug Administration has granted modified risk tobacco product (MRTP) status to eight Premarket Tobacco Application (PMTA) authorised General snus products, pointing to the viability of Swedish snus as reduced risk alternatives for adults who would otherwise continue to smoke. Oral nicotine pouches (ONPs) are similar to snus but differ by being tobacco-free (containing nicotine but not tobacco leaf thereby suggesting that exclusive ONP use may present less health risks than smoking cigarettes and comparable or even less risk than using snus. The aim of this study was to conduct a comprehensive analysis of toxicant levels in ONPs compared with Swedish snus.

**Measurements:**

Using standard and verified methods, 132 analytes were measured in 11 commercial ONPs (including nine Velo products), a non-commercial, unflavoured zero-nicotine content ONP, and two snus products (1S4 reference snus and a commercial snus) to better understand each product’s toxicant profile.

**Findings:**

In total, the contents of 70 analytes were not quantifiable in any of the analysed ONPs or snus. A higher number of analytes were present at quantifiable levels in snus (*n* = 58) than in ONPs (*n* = 47) on a per-pouch basis. Of the 43 analytes that were above the detection limit in both product types, 15 analytes were quantifiable only in snus, and four were quantifiable only in some ONPs. In most tests, the ONPs contained lower levels of analytes (> 90% lower) than the snus study products. Comparing the chemical contents of ONPs with historical cigarette smoke data showed almost all comparators were substantially lower in ONPs. Regarding the subset of analytes listed in the GOTHIATEK^®^ standard, none of the ONPs tested exceeded the maximum allowable limits established for snus.

**Conclusion:**

The results support the hypothesis that exclusive use of ONPs might offer a potentially reduced risk alternative relative to continued smoking and comparable or even less risk than using snus.

**Supplementary Information:**

The online version contains supplementary material available at https://doi.org/10.1186/s13065-026-01895-x.

## Introduction

Traditionally, consumer products that contain nicotine have been tobacco-based. These include combusted inhalation products (such as cigarettes, cigars and pipe tobacco), or oral smokeless tobacco products (STPs, such as snus, snuff and chewing tobacco). However, when tobacco is combusted, its use can be harmful. Inhaling tobacco smoke can induce respiratory diseases, including lung cancer and chronic obstructive pulmonary disease [1]. This morbidity and mortality arises substantially from consumer exposure to a range of carcinogens and other toxic compounds, collectively referred to as Harmful and Potentially Harmful Constituents (HPHCs) [2] or toxicants [3] rather than from the respective products’ nicotine contents [4, 5].

Observation of population-scale disease patterns in different countries led to the conclusion that some tobacco products pose lower risks than others [6, 7] due to differences in toxicant content. For example, the Royal College of Physicians [6] concluded that some STPs, such as Swedish snus and American chewing tobaccos, present lower health risks than others, consistent with differences in their toxicant content, supporting the concept of ‘tobacco harm reduction’ proposed by the US Institute of Medicine [8].

Tobacco harm reduction (THR) is a public health approach aimed at reducing the harm caused by tobacco use. It focuses on encouraging adult smokers who would otherwise continue smoking to switch completely to smokeless alternatives that are potentially reduced risk relative to continued smoking. Swedish snus, a moist oral STP manufactured with specific tobacco blends using processes designed to produce a comparatively low toxicant profile [9], has been identified in the past decade as an established contributor to THR [10–13]. In addition to its low toxicant content, use of oral products such as snus avoids the combustion products and direct lung exposure to HPHCs associated with inhaling tobacco smoke. Extensive epidemiological data shows that use of Swedish snus confers reduced health risks as compared to cigarette smoking. These factors have contributed to the US Food and Drug Administration (FDA) granting a modified risk order for eight Swedish snus products [13].

In the past few decades, new nicotine-containing products with a reduced toxicant profile as compared to cigarettes and other tobacco products have emerged. The first such nicotine products were nicotine-replacement therapies (NRTs) in the form of patches, gums, lozenges and inhalers [14, 15]. These products were specifically designed to help smokers quit. E-cigarettes [16] and tobacco heating products [17] have gained traction among consumers as popular smokeless alternatives to conventional cigarettes.

More recently, oral nicotine pouches (ONPs) have been brought to market that may be potentially less harmful to health than many of the traditional STPs used globally. These pouches are similar in design and use to Swedish snus but are tobacco-free. ONPs are placed between the user’s upper lip and gum [18] to allow nicotine uptake through the oral mucosa. They contain a non-tobacco substrate (e.g., microcrystalline cellulose and water), pharmaceutical-grade nicotine, pH buffers, filling agents, humectants, salt, food-grade flavourings, taste additives and sweeteners inside a permeable viscose fibre pouch [18–20]. While snus products contain many similar components to ONPs, such as salt, water, sodium carbonate (pH buffer), humectants and food-grade flavourings [9, 21], snus is a tobacco product, containing a blend of air- and sun-cured tobaccos. The presence of tobacco means the chemical composition of snus is intrinsically more complex than that of ONPs.

ONP use has rapidly grown in popularity, particularly in countries with a history of snus-use. For example, recent population-survey studies in Sweden [22] have reported that young people have predominantly switched from use of snus to ONP-use, with “white snus” the product now mainly used nationally by these consumers. In both Norway and Sweden, nicotine pouch sales (on-line retail) have grown as snus sales (on-line retail) have declined, with ONP/white snus sales exceeding snus sales in both countries in 2025 [23]. In Iceland, ONP use has grown significantly, and population surveys suggest that its prevalence rivals that of snus [24]. ONP use has also increased amongst smokers [25], but there is currently insufficient data to characterise the efficacy of ONPs for smoking cessation or reduction in the number of cigarettes smoked per day.

McNeill and Munafo [26] developed the concept of a risk continuum of tobacco and nicotine products based on the principles of THR, coupled with an understanding of Swedish snus’s historical role in reducing the population harm associated with smoking in Sweden and the emergence of alternative nicotine products. On this continuum, combustible cigarettes pose the greatest risk to a user’s health while NRTs pose the least risk. Swedish snus poses a risk close to NRTs at the lower risk end of the continuum.

Considerable efforts are being expended toward establishing the position of new nicotine products, such as ONPs, on the risk continuum. The potential THR benefits of alternative nicotine products may take decades to confirm epidemiologically. Consequently, assessment strategies have been developed to provide early insights into risks associated with use of alternative nicotine products [7, 27, 28]. An important stage in such risk characterisation is non-clinical assessment, including laboratory-based steps such as chemical analysis of the toxicant contents of products.

Several chemical analyses of ONPs have been published [20, 29–34], focusing primarily on the toxicants most frequently used to characterise STPs: namely, the FDA’s notice of Harmful and Potentially Harmful Constituents in Tobacco Products and Tobacco Smoke; Established List [2] and the GOTHIATEK^®^ standard which is a voluntary quality standard developed by Swedish Match setting maximum allowable levels for substances like nitrosamines (NNN, NNK), heavy metals (lead, cadmium, arsenic, mercury, nickel and chromium), agrochemical residues, aldehydes (formaldehyde, acetaldehyde and crotonaldehyde) and mycotoxins (aflatoxins and ochratoxin A) [9]. Overall, these studies have shown that ONPs have comparable or lower levels of a narrow range of toxicants relative to snus products. For example, Wagner et al. [29] analysed the contents of nine toxicants in several variants of ONPs and observed either no detectable levels or significant reductions (apart from nicotine) as compared with cigarettes and STPs, including snus products. Of the other published studies, Back et al. [33] analysed the broadest range of toxicants, comprising 36 HPHCs plus two additional tobacco-specific nitrosamines (TSNAs) and five further compounds subject to maximum levels under the GOTHIATEK^®^ standard. Studied nicotine products included a commercial ONP, snus, moist snuff and pharmaceutical NRTs. They detected no quantifiable levels of nitrosamines or polycyclic aromatic hydrocarbons (PAHs) in, and comparable number of quantifiable HPHCs between the tested ONP, and NRTs.

Given their longer history of use, there are many more studies of the chemical composition of STPs than ONPs in the scientific literature [35]. Perhaps the most complete chemical analysis of STPs arises from the studies undertaken by McAdam et al., who investigated the contents of 117 measurands in more than 70 contemporary STPs, including dry and moist snuffs, US chewing tobaccos, and pouched and loose Swedish snus. Individual studies focused on organic toxicants, including carbonyls, hydrocarbons and aromatic amines [36], toxic trace elements [37], volatile and involatile nitrosamines and TSNAs [38], nitrosoacids [39] and diverse TSNAs [40], polycyclic aromatic hydrocarbons [41], hydrazine [42], acrylamide [43], radioactive elements [44], coumarin and angelica lactones [45], ethyl carbamate [46], and the major non-toxicant STP components [21], as well as microbiological activity and associated toxicants [38].

The main goal of the present study was to extensively characterise the chemical composition of ONPs compared with snus, and to compare these data to the composition of cigarette smoke. These data are an important first step toward positioning ONPs on the tobacco and nicotine risk continuum [26]. Such data also make an important contribution to understanding changes in exposure to toxicants that might result from switching from snus to ONPs, given the population-level changes in usage patterns of these products reportedly occurring in Nordic countries.

The present study focused on the 117 toxicants previously examined in STPs noted above. These comprised the carcinogens and chemical agents identified in major literature reviews, summaries and guideline lists of toxicants present in STPs [9, 47, 48], and were extended to include additional toxicants previously identified in tobacco [49] and related matrices [44].

The list of 117 analytes measured historically for STPs included relevant GOTHIATEK^®^ analytes [9], the priority FDA abbreviated smokeless tobacco list of Harmful and Potentially Harmful Constituents (HPHCs [50, 51]), and included the FDA established list of 93 HPHCs for tobacco products or tobacco product smoke [2] (which was extended by a further 18 HPHCs in April 2026 [52]), but excluding those established list HPHCs identified as not relevant to smokeless tobacco products [53].

For the present study, the list of 117 toxicants previously measured in STPs was extended to include 15 additional species reported in recent publications on ONP chemistry. In total, a comprehensive list of 132 measurands was analysed in 11 commercial ONPs varying in flavour, nicotine content, and manufacturer. In addition, a non-commercial unflavoured, zero-nicotine content ONP was included for validation purposes. Two snus products were also evaluated to provide both a known comparator base and a bridge to the broader range of STPs examined previously.

## Methods

### Study samples

The study samples, including brand, manufacturer and nicotine content, are summarized in Table 1. 11 ONPs were commercially available at the time of the study and were sourced from the marketplace in 2022; they comprised nine products manufactured by Nicoventures Trading Limited under the brand name “Velo”, and two competitor products (labelled CP1 and CP2). A twelfth (non-commercial) ONP, an unflavoured, zero-nicotine product formulated by Nicoventures Trading Limited with the same material composition as the flavoured Velo nicotine products, was included in the study for method evaluation. The two snus products were the Swedish-style snus reference product, 1S4 (University of Kentucky, Lexington, NY, USA), sourced from the University of Kentucky and the market-leading product in Sweden, General Classic Blend White Portion Large Snus (Swedish Match, Stockholm, Sweden), sourced from the marketplace in 2022. After acquisition, all study products were stored at − 20^o^ C before analysis. For comparison with cigarette smoke, published data on the reference cigarette 1R6F [17] was used.

### Analytical methodologies

All analyses reported in this study were conducted at Independent Contract Laboratories. These comprised: Eurofins CRL, Piscataway, NJ, USA; Eurofins Food & Feed Testing (FFT), Lidköping, Sweden; Labstat International, Kitchener, Ontario, Canada and three laboratories based in the UK.

Laboratories were selected based on their availability of analytical methods for the specific analytes in this study, whether in Snus, ONPs, or vegetable materials. The availability of fully validated analytical methods for Snus and/or ONPs was also a primary consideration. All samples were measured for an analyte group in a single laboratory. 30 analytical methods were used for the 132 measurands in the present study. These methods are summarised in Table 2, and reported in greater detail in Supplementary File 1, Section S1.

### Method validation

In terms of method validation for the matrices of interest (snus and ONPs), the following process was followed:

- Where independent laboratory analytical methods existed for an analyte (almost all analytes), we used laboratories with methods already validated for both snus and ONPs (Table 2). No further validation was conducted for these methods in our study.
- Where a validated external laboratory method existed for snus but not ONPs (Table 2), the validated snus method was tested for suitability with ONPs using the procedure described in Supplementary File 1, Sect.  2 of the revised manuscript. The rationale for assessing the suitability of methods validated for snus on ONP samples was the physical similarity between the two matrices (albeit with ONPs having relative compositional simplicity compared to snus). The results of the validation steps for these methods are also given in Supplementary File 1, Sect.  2.
- Where no method was available from external laboratories for an analyte group with either snus or ONPs, we commissioned the development of a new method (Table 2) and evaluated it for both matrices. Outcome summaries of the validation steps for these methods are given in Supplementary File 1, Sect.  3.

During the measurement exercise, three replicates were measured for each analyte, except for the radionuclides (single replicate) and mass (36 replicates). For the radionuclide analysis, a single replicate was conducted on a composite sample of approximately 24 g per sample, comprising one or more containers of ONPs or snus. With the measurement of product masses, each laboratory measured mass as part of their measurement programme. In total, thirty-six replicates of mass measurements were returned from the external laboratories for each product; all of these values were used to calculate the mean product masses.

All analyses were conducted on the entire ONP or snus pouch (i.e. the fleece and contents together).

### Method sensitivities

Limits of detection (LOD - the lowest concentration level that can be determined to be statistically different from a blank), limits of quantification (LOQ - the level above which quantitative results may be obtained with confidence) and reporting limits (RL - the smallest concentration of an analyte that a laboratory can reliably report) are presented for all methods in Supplementary File 2, Table S1. Most laboratories reported LODs and LOQs, however in some cases, neither LOD nor LOQ was reported, an RL was provided instead. LODs and LOQs were also reported differently by the laboratories: some reported LOD/LOQ per pouch; others reported LOD/LOQ per gram of material, and with one analyte content was expressed as a %. We converted the values to obtain RL/LOD/LOQ per pouch and RL/LOD/LOQ per gram (using measured pouch weights) to provide a complete picture for data analysis.

#### Source apportionment of PAH contents

To identify the source of any PAHs detected in the study products, ratios of benzo[a]anthracene/(benzo[a]anthracene+chrysene), Indeno[1,2,3-cd]pyrene/benzo[a]pyrene (B[a]P), anthracene/(anthracene+phenanthrene), fluoranthene/(fluoranthene + pyrene), benzo[k]fluoranthene/ benzo[a]pyrene and benzo[b]fluoranthene/benzo[a]pyrene were calculated and compared with reference values for different PAH sources [41].

#### Study product comparisons

All data analyses were conducted using statistical analysis system (SAS) System for Windows Version 9.4 (SAS Institute, Cary, NC, USA). The analyte contents of ONPs and snus samples were compared in two ways: per-pouch and per-gram. The per-pouch basis was considered the most robust comparison because it allowed a like-for-like comparison of potential exposure in terms of a snus user swapping STPs to ONPs. The per-gram basis data was included to facilitate additional analysis considering the differing masses of the various ONP and snus pouches.

The focus of the study product comparisons reported in this study were between the 11 commercial ONPs and the two snus products. The twelfth ONP, the zero-nicotine, unflavoured sample is not used by consumers, unlike the 11 commercial ONPs. It was included in some, but not all analyses, as a comparator and for method validation purposes. Due to its lack of relevance to consumer exposure, and the incomplete chemical dataset it was not included in the quantitative comparison calculations between snus and ONPs. Separate comparisons were made between the 11 commercial ONPs and the commercial snus product (General Classic White Blend, portion), and between the 11 ONPs and reference snus product 1S4. The purpose of the 1S4 reference product is to provide the physical and chemical characteristics of typical Swedish snus products. These latter comparisons with 1S4 allows for wider and more generic comparisons between ONPs and snus than would be provided by a comparison to a single commercial product.

Mean and standard deviation were determined for each measurand and product sample for both per-pouch and per-gram analyses. Differences between products are reported as percentage differences from snus products to ONPs, calculated by the following equation:

$$\begin{gathered} {\mathrm{Percentage~difference}} \hfill \\ =\frac{{\left( {{\mathrm{ONP}}~{\mathrm{sample~mean}} - {\mathrm{Snus~sample~mean}}} \right) \times 100}}{{{\mathrm{Snus~sample~mean}}}} \hfill \\ \end{gathered} $$  

For analytes that were either < LOQ, < LOD or < RL, accurate comparisons were not possible therefore, imputed values were used to estimate percentage differences between samples. In-line with previous studies [16, 54, 55], the following steps were taken:

- The percentage difference was not calculated for measurands that were < LOQ in both the ONP and snus.
- For measurands that were < LOQ, < LOD or < RL in the ONP, but quantifiable in the snus comparator, the percentage difference was estimated by imputing a value for the ONP as follows. For ONP data < LOD or < RL, the analyte content of the ONP was estimated as one-half of the analytical method’s reported LOD or RL. For data < LOQ but > LOD, the value was calculated as the midpoint between the reported LOD and LOQ of the analytical method (i.e., imputed value = 0.5 × [reported LOQ − reported LOD] + reported LOD). It has been demonstrated that errors in estimates for the numerator introduced by this calculational approach are small [48]).
- For measurands that were < RL, < LOQ or < LOD in snus, but quantifiable in the ONP sample, the percentage difference was estimated using the RL, LOQ or LOD value for the snus sample. Calculations of this kind provide estimates of minimum differences; thus, the results are reported in the form “> x%”, where x% is the minimum difference value generated using the RL, LOQ or LOD. This approach avoids the possibility of introducing significant errors in the calculation through division by an uncertain denominator (for the snus content).

For the plots of percentage difference in chemical contents between ONP and snus product categories, the % difference was calculated using a mean value for all ONP contents (calculated as the mean of *n* = 11 or *n* = 8, depending upon the analyte) in comparison to the mean value for the two snus samples. This provided a single percentage difference value for each analyte.

For comparison with cigarette smoke, published data on the reference cigarette 1R6F [17] was used. Percentage differences were calculated between the analyte values per cigarette (1R6F) and the analyte values per pouch (ONPs). Toxicant contents for the ONPs were averaged across all 11 commercial ONPs to provide a single comparator to cigarette smoke. The same approach was used when comparing snus products with cigarette smoke.

In addition to the general comparison, we focused on priority toxicants previously identified by the WHO Tobacco Product Regulation Working Group (TobReg), including nine key toxicants mandated for reduction in cigarette smoke and considered to be high priority for disclosure and monitoring [56]. Percentage differences between snus products and cigarette smoke, and between ONPs and smoke were examined for these toxicants.

## Results

To characterise the chemical contents of ONPs and contribute to the scientific evidence of their position on the tobacco and nicotine risk continuum, the present study evaluated 11 commercial ONPs, one non-commercial, zero-nicotine unflavoured ONP and two snus products for 132 measurands previously measured in either snus products or ONPs.

Where available (Table 2), validated methods were used to measure snus and ONP contents. Where validated snus methods were used to measure ONP contents, additional validation steps were conducted using ONP samples.

Prior to analysis of the study products, the various methods were validated for use with ONP samples and showed acceptable performance for all analytes (Supplementary File 1 Section S2). LODs, LOQs and RLs for the analytical methods used in the present study are provided in Supplementary File 2, Table S1.

Detailed sample-by-sample data for the 132 measurands is provided in Supplementary File 2, Table S2, which lists the measured chemical contents for all 14 study products, while Table 3; Fig. 1 summarise the analytes present at quantifiable levels in ONPs only, in snus products only, in both product types or in neither. Supplementary File 2 Tables S3 and S4 list the percentage differences in the measurands between commercial ONPs and the 1S4 reference snus, and between ONPs and the commercial snus, General Classic Blend White Portion Large Snus, respectively.

As shown in Fig. 1, fewer measurands were present in ONPs at quantifiable levels than snus products (Fig. 1). Overall, 43 measurands were quantifiable in both product types, 15 were quantifiable only in snus products, and four only in one or more ONPs. Many of the measurands common to both ONPs and snus products were only present in a minority of ONPs (Fig. 1). Below we describe key observations from the measurements and differences between ONPs and snus products.

### Major measurands

#### Sample mass

For 9 of the 11 commercially available ONPs, pouch mass was close to 700 mg with a coefficient of variation (within sample) of less than 9.5%. One ONP (Velo Salt Freeze 20 mg) had a substantially greater mean mass per pouch of 1285 mg, and one (CP1) had a substantially lower mass of 260 mg. In contrast, both snus products were heavier than most ONPs, having mean masses of 889.4 and 999.7 mg per pouch.

#### Water content

The water content in ONPs varied widely from 4.1 to 49.4%. All ONPs, except one product, had water contents of 33.6 to 49%, while the two snus samples had a water content of around 49%. The ONP with the low water content (4.1%) was CP1 Mint 8 mg.

#### pH

All of the ONPs had higher pH values (range 8.5 to 9.1) than the two snus samples (7.4 and 8.1).

### Hydrocarbons

#### Aliphatic and aromatic species

In total, five volatile aliphatic and aromatic hydrocarbons were included as part of the volatile organic compound analyses. Of these, benzene, isoprene and 1,3-butadiene were not detected in any sample. Styrene was detected (< LOQ) in four ONPs (two Velo Tropic Breeze samples, CP1 and CP2) and the commercial snus product but not detected in the other seven ONP samples and the reference snus product.

Toluene was found at quantifiable levels in both snus samples and three ONPs (a Velo Tropic Breeze product, CP1 and CP2), detected (< LOQ) in two other ONPs (the unflavoured product and the other Velo Tropic Breeze product), and not detected in the other six ONPs, on both a per-pouch and per-gram basis. All ONPs had lower toluene content per pouch than the reference snus (mean, 76% lower; range, 21 to 93% lower) and nine ONPs had lower content than the commercial snus (overall mean, 51% lower; range, 61% higher to 86% lower). Expressed per gram, the toluene content was, on average, 59% lower (range, 15% higher to 94% lower) in ONPs than in the reference snus product and 31% lower (range, 94% higher to 91% lower) in ONPs than in the commercial snus product with corresponding data presented in Supplementary File 2, Table S2.

#### Polycyclic aromatic hydrocarbons

Supplementary File 2, Table S2, shows that 18 of the 21 PAHs measured were present at quantifiable levels in the two snus samples. In contrast, only eight of the 21 PAHs were quantifiable in all ONPs, and these eight PAHs were also quantifiable in the snus samples. The eight PAHs were acenaphthene, acenaphthylene, benzo[g, h,i]perylene, fluorene, naphthalene, 1-methylnaphthalene, 2-methylnaphthalene, and phenanthrene.

Of the three PAHs not quantified in either snus sample, perylene was not detected in any ONP or snus samples. Benzo[a]pyrene was not detected in either snus sample or in nine of the ONPs but was present at levels below LOQ in three ONPs (the unflavoured product, and two Velo products – Hybrid Urban Vibe and Salt Freeze). Benzo[e]pyrene was detected (< LOQ) in three ONPs (the same products as with Benzo[a]pyrene) and the commercial snus product.

The acenaphthene content per pouch was lower in nine ONPs than in the commercial snus product; mean levels across all ONPs were 20% lower per pouch. On a per-gram basis, five of the ONPs had higher acenaphthene content than the commercial snus product, and six ONPs had lower content (mean across all ONPs was 7% higher). Relative to the reference snus product, four ONPs had higher acenaphthene content and seven had lower content per pouch. Mean per-pouch content for ONPs was 18% higher. On a per-gram basis, six ONPs had higher acenaphthene content than the reference snus product (mean for all ONPs, 46% higher).

Regarding acenaphthylene, nine ONPs had lower per-pouch content, seven and eight had lower per-gram content compared with the reference and commercial snus products, respectively. The mean acenaphthylene content in the ONPs was 2% and 26% lower per pouch, and 32% and 6% higher per gram than in the reference and commercial snus products, respectively.

The benzo[g, h,i]perylene content of 10 ONPs was lower than that of the two snus products (mean of the 10 ONPs, 74% lower per pouch and 63% lower per gram vs. reference snus product; and 67% lower per pouch and 50% lower per gram vs. commercial product). However, one ONP (Velo Sweet Polar Mint, 10 mg) had a benzo[g, h,i]perylene content higher than that in snus (145% higher per pouch and 225% higher per gram vs. reference snus product, and 210% higher per pouch and 341% higher per gram vs. commercial snus product).

Relative to the two snus samples, the fluorene ONP contents were lower in eight ONPs per pouch and seven ONPs per gram. Overall, however, the mean ONP fluorene contents per pouch were 9% and 31% higher than the commercial and reference snus products, respectively. Per-gram the ONPs were on average 30% and 45% higher in fluorene content than the commercial and reference snus products, respectively.

As compared with the reference and commercial snus products, six to seven ONPs, respectively, had lower 1-methylnaphthalene content on a per-pouch basis. The mean ONPs’ content had 7% lower and 11% higher levels per pouch, compared to the commercial and reference snus products, respectively and 44% and 59% higher levels per gram compared to the commercial and reference snus products, respectively.

Six ONPs had lower 2-methylnaphthalene content per pouch than the two snus products and two ONPs had lower content per gram than the commercial snus product. Overall, the mean 2- methylnaphthalene contents of the ONPs were 84% and 137% higher per pouch and 179% and 236% higher per gram than the commercial and reference snus products, respectively.

Regarding naphthalene, seven to eight ONPs had lower content per pouch and three to six ONPs had lower content per gram than the reference and commercial snus products, respectively. Overall, naphthalene contents of the ONPs were 3% and 19% lower per pouch and 34% and 19% higher per gram than the reference and commercial snus products, respectively.

For phenanthrene, compared with the reference snus, all ONPs were lower per gram than the reference snus; levels per pouch were lower in all but one ONP (Velo Salt Freeze 20 mg), and mean ONP contents were 76% and 80% lower, respectively. All ONPs had lower phenanthrene contents than the commercial snus product (mean values were 85% lower per pouch and 81% lower per gram).

The remaining 10 PAHs were quantified in the two snus products. Of these, benzo[j]fluor-anthene was not detected in 10 ONPs and was detected but not quantified for one ONP (Velo Salt Freeze); the levels were 98% lower per pouch and 97% lower per gram than those of both snus products. Benzo[k]fluoranthene was not detected in 10 ONPs and quantified in one ONP (Velo Salt Freeze); overall mean ONP levels were 94% lower per pouch and 94% lower per gram than both of the snus products. Benzo[a]anthracene was quantified in two ONPs (Velo Salt Swedish Mint and CP2), detected but not quantified in eight ONPs, and not detected in one ONP (Velo Sweet Tropic Breeze 10.9 mg). On average, the levels were 95% lower per pouch and 93% and 94% lower per gram in the ONPs than the commercial and reference snus products, respectively.

Seven ONPs contained quantifiable levels of fluoranthene and indeno[1,2,3-cd]pyrene. Overall, the levels of fluoranthene were 97% lower on a per-pouch and per-gram basis in ONPs than in the two snus products, while those of indeno[1,2,3-cd]pyrene were 87% and 83% lower per pouch and 83% and 75% lower per gram than the reference and commercial snus products, respectively. Six ONPs contained quantifiable levels of benzo[b]fluoranthene; the mean level of this PAH was 92% and 93% lower per pouch and 88% lower per gram in the ONPs than in the reference and commercial snus products, respectively.

Four ONPs contained quantifiable levels of pyrene and anthracene, chrysene and dibenz[a, h]anthracene. Anthracene and pyrene were quantifiable in the same four ONPs (Unflavoured, and Velo Hybrid Urban Vibe 10.9 mg, Salt Swedish Mint 6 mg, and Salt Freeze 20 mg). Chrysene and dibenz[a, h]anthracene were both quantifiable in three of the same ONPs (CP2, Velo Salt Swedish Mint 6 mg, and Hybrid Tropic Breeze 6 mg), as well as one other ONP each.

The pyrene content of the ONPs was on average 96% lower per pouch and 95% lower per gram than that of the two snus products; the anthracene content was 84% and 87% lower per pouch (although one ONP had a higher content than the reference snus sample) and 84% and 86% lower per gram than the reference and commercial snus products, respectively; the chrysene content was 96% and 97% lower per pouch and 95% lower per gram than the reference and commercial snus products, respectively; and the dibenz[a, h]anthracene content was 73% and 74% lower per pouch and 58% and 56% lower per gram than the reference and commercial snus products, respectively.

### Oxygenated analytes

#### Carbonyls

The study included four carbonyls in the analysis. Of these, crotonaldehyde was not detected in any ONP; it was detected (< LOQ) in the commercial snus sample, but not in the reference snus sample.

Acetaldehyde was quantified in six ONPs, detected (< LOQ) in the other ONPs, and quantified in both snus products. All ONPs had lower acetaldehyde content per pouch (mean, 99% lower; range, 98 to 99%) and per gram (mean, 98% lower; range, 94 to 99% lower) as compared with the reference snus product. Relative to the commercial snus product, the ONPs contained, on average, 99% per pouch (range, 97 to 99%) and 98% per gram (range, 94 to 99%) lower levels of acetaldehyde.

Acrolein was measured at quantifiable levels in two ONPs (CP1 and CP2) but not detected in the other 10; it was detected (< LOQ) in the commercial snus product but not the reference snus product. On a per-pouch basis, the two ONPs with quantified contents had acrolein levels > 299% and > 814% higher than those of the reference snus product, and > 20% and > 175% higher than those of the commercial snus product. On a per-gram basis, the two ONPs with quantified acrolein content had approximately > 490% and > 3957% higher levels than the reference snus product, and > 41% and > 866% higher levels than the commercial snus product.

Formaldehyde was quantifiable in all ONPs and the two snus products. On a per-pouch basis, two ONPs had higher formaldehyde content (Velo Hybrid Urban Vibe 10.9 mg at 15% higher and Velo Sweet Polar Mint 10 mg at 130% higher), and the other ONPs had lower formaldehyde content (range, 13 to 71%) than the reference snus product. Overall, the mean formaldehyde content per pouch was 22% lower in the ONPs than in the reference snus product. In comparison to the commercial snus product, three ONPs had higher per-pouch formaldehyde content (the two products, above, with higher formaldehyde content than the reference product plus Velo Sweet Polar Mint 6 mg, mean, 67%; range, 1 to 167%); the other ONPs had lower contents (mean, 39%; range, 20 to 67%). Overall, the average formaldehyde content per pouch was 10% lower than that of the commercial snus product.

On a per-gram basis, five ONPs had higher formaldehyde content relative to the reference snus product (mean, 88%; range, 5 to 235%), and the others had lower content (mean, 28%; range, 2 to 76%); the mean formaldehyde content per gram was 25% higher in the ONPs than in the reference snus product. The mean per-gram formaldehyde content was 20% higher in the ONPs than in the commercial snus product; five ONPs had higher levels (mean, 80% higher; range, 1 to 222% higher); the other ONPs had lower levels (mean, 31% lower; range, 6 to 77% lower). The mean formaldehyde content per gram was 20% higher in the ONPs than in the commercial snus product.

#### Miscellaneous oxygenated species

Among other oxygenated compounds, coumarin, and the α- and β-angelica lactones were not detected in any of the ONPs or snus products. The contents of both total and reducing sugars was below the LOQ in all study products. In addition, none of the ONPs or snus products contained measurable levels of aflatoxin B1, B2, G1 or G2.

Glycerol was detected at quantifiable levels in 1 ONP (CP2) but not detected in the other ONPs or in either snus product. Compared with the two snus products, this ONP had an estimated glycerol content > 9000% higher per pouch and > 10,000% higher per gram.

Propylene glycol was found at quantifiable levels in both snus products and six ONPs; it was detected but not quantified in two further ONPs. Relative to both reference and commercial snus products, ONPs had a mean propylene glycol content that was 85% lower per pouch (range, 50 to 99%) and 76% lower content per gram (range, 28 to 99%).

### Microbiological activities

Total bacterial counts were measurable only for the reference snus (41.5 CFU per pouch) and not for any of the ONPs or the commercial snus product.

All ONPs had mould contents below the RL, as did the commercial snus product. The total yeast content was below the RL for all products in this study.

### Nitrogenous analytes

#### Alkaloids

*Total alkaloids* All samples contained quantifiable levels of total alkaloids, as presented in Supplementary File 2, Table 2. On a per-pouch basis, seven ONPs had higher total alkaloid content than both the reference snus product (mean, 75% higher; range, 1 to 302% higher) and the commercial snus product (mean, 112% higher; range, 22 to 387% higher). The other ONPs had lower total alkaloid content on a per-pouch basis, than the reference snus product (mean 29% lower; range, 21 to 35% lower) and the commercial snus product (mean, 15% lower; range, 5 to 22% lower). Overall, the ONP total alkaloids content as a mean was 37% and 66% higher per pouch and 157% and 176% per gram than that of the reference and commercial snus products, respectively.

*Nicotine and related compounds* Apart from the unflavoured zero-nicotine ONP, all samples contained quantifiable levels of nicotine. Similar to total alkaloids, some ONPs had higher nicotine contents (seven to eight ONPs on a per-pouch basis and 11 ONPs on a per-gram basis) than those of the commercial and reference snus products. The remaining minority of ONPs had lower nicotine content than the commercial and reference snus products. Overall, the mean nicotine content was 22% and 49% higher in the commercial ONPs than in the reference and commercial snus products on a per-pouch basis, respectively. On a per-gram basis, the nicotine content was, on average, 78% and 92% higher in the commercial ONPs than in the reference and commercial snus products, respectively. The ONPs had a nicotine content within 20% of (and predominantly closer to) the stated pack value.

All seven nicotine-related compounds were quantified in the two snus products. Nornicotine and nicotine-1-oxide were quantifiable in all ONPs and were present at lower per-pouch levels than in both snus products. Regarding nornicotine, the ONPs had significantly lower content (range, 96 to 93% lower) on both a per-pouch and per-gram basis relative to the two snus products. Nicotine-1-oxide content was 66% lower per pouch, 49% and 58% lower per gram in the commercial ONPs than in the reference and commercial snus products, respectively.

Myosmine and cotinine were quantifiable in all ONPs other than CP1. The mean myosmine content was 69% and 81% lower in the commercial ONPs than in the commercial and reference snus products on a per-pouch basis, respectively. The cotinine content of the commercial ONPs was, on average, 98% lower than that of the snus products both on a per-pouch and per-gram basis.

β-Nicotyrine was quantified in four of the commercial ONPs, and anatabine and anabasine were quantified in three. Mean β-nicotyrine content was 98% lower on a per-pouch basis, and 97% and 98% lower on a per-gram basis in commercial ONPs than the reference and commercial snus products, respectively. The anatabine content was, on average, 98% and 97% lower in commercial ONPs than in the reference and commercial snus products, respectively, while the anabasine content was 94% and 93% lower. These reported differences include both per-pouch and per-gram calculations.

#### Aromatic amines

The four analysed aromatic amines were not detected in any ONP sample. 1-Amino naphthalene and 2-amino naphthalene were not detected in either snus product; however, 3-aminobiphenyl was detected but not quantifiable in the commercial snus product, and 4-aminobiphenyl was detected (< LOQ) in both of the snus products.

#### Tobacco specific nitrosamines (TSNAs)

Four of the seven measured TSNAs (NAB, NAT, NNK and NNN) were not detected in any of the ONPs but were quantified in both snus products. The levels of the four TSNAs were estimated to be 97 to 99% lower in the ONPs than in both the snus products, whether expressed on a per-pouch or per-gram basis. The other three TSNAs, NNAL, iso-NNAL and NNA, were not quantifiable in any study product.

#### Volatile and involatile nitrosamines

Of the 12 volatile and involatile nitrosamines analysed, eight were not detected in any of the ONPs or either snus products, including all of the di-aliphatic nitrosamines (NDMA, NEMA, NDEA, NDPA, NDBA and NDiPA), and the two unsaturated cyclic nitrosamines (NPYR and NMOR).

NDBzA was detected at quantifiable levels in one ONP, but it was not detected in the other ONPs or either snus product. The NDBzA content of this ONP was higher on a per pouch basis, 3.3 ng and on a per gram basis 4.9 ng than that of the snus products (< 0.177ng per pouch and < 0.1907 per gram). NPIP was detected (< LOQ) in eight ONPs and both snus products, but not in the other three ONPs.

Both snus products contained quantifiable levels of NDELA, as did five commercial ONPs and the unflavoured zero-nicotine ONP. This compound was also detected (< LOQ) in the other six ONPs. The NDELA content was 29 to 76% lower in the commercial ONPs than in the reference snus product per pouch, and 97% higher to 82% lower in the ONPs per gram. As compared with the commercial snus product, the commercial ONPs had 16 to 71% less NDELA per pouch and 112% higher to 81% lower NDELA per gram. NDiPLA was not detectable in either snus product or five of the 11 commercial ONPs but was detected (< LOQ) in the other six ONPs.

#### Nitrosoacids

The study analysed 11 nitrosacids in the study products. For four products, analysis of *N*-nitroso-l-hydroxyproline was unsuccessful due to equipment failure. Overall, nine of the 11 acids were not measurable in any sample. The other two, *N*-nitroso-l-proline and 3-(methylnitrosamino) propionic acid, were detected at reporting levels in both snus samples, but not in any of the ONPs.

The per-pouch 3-(methylnitrosamino) propionic acid content of the commercial ONPs was estimated to be 77% and 80% lower than that of the reference and commercial snus products, respectively on a per pouch basis. On a per-gram basis, ONPs had 55% and 66% lower content than the reference and commercial snus, respectively.

The *N*-nitroso-l-proline contents of the ONPs were, on average, 93% per pouch lower than the reference snus and 85% lower than the commercial snus products. On a per-gram basis, the ONPs had on average, 90% and 82% lower levels than the reference and commercial snus products, respectively.

#### Miscellaneous nitrogenous analytes

Acrylamide was detected at quantifiable levels in five ONPs but was not quantifiable in the other ONP samples. It was also present at quantifiable levels in both snus products. Relative to the snus products, the ONPs contained a mean of 92% (range, 86 to 97%) less acrylamide than the reference snus product and a mean of 89% (range, 81 to 96%) less acrylamide than the commercial snus product on a per-pouch basis. On a per-gram basis, the ONPs had on average, 88% and 86% lower levels than the reference and commercial snus products, respectively.

Acrylonitrile was not detected in any ONP or snus product.

Ethyl carbamate was present at quantifiable levels in three ONPs (Velo Hybrid Tropic Breeze 6 mg, and both 6 mg and 10.9 mg Velo Sweet Tropic Breeze products), and the reference snus product. It was not detected in seven ONPs or in the reference snus product. In comparison to the reference snus product (where ethyl carbamate was not detected, < 0.789 ng per pouch and < 0.8134 ng per gram), the ONPs containing quantifiable levels had higher ethyl carbamate per pouch 35.37 to 66.83 ng, and per gram 49.36 to 96.01ng. Relative to the commercial snus product (where ethyl carbamate was quantified at 13.53 ng per pouch and 15.07 ng per gram), the ONPs with no detectable ethyl carbamate had 98% and 97% lower content (per pouch and per gram), while the three ONPs with quantified levels had 161 to 394% higher content per pouch and 228 to 537% per gram.

Hydrazine was detected in one ONP, but at levels too low to quantify. Hydrazine was not detected in any other analysed sample.

Regarding nitrate, both snus products had quantifiable levels, whereas all ONPs had nitrate content < LOQ. On average, the ONPs had nitrate levels that were 91% and 93% lower per pouch than the commercial and reference snus products, respectively. On a per-gram basis, the nitrate content of the ONPs was, on average, 89% and 90% lower than that of the commercial and reference snus products, respectively.

### Inorganic species

#### Ash

All of the measured study products contained quantifiable levels of ashable inorganic content as reported in Supplementary File 2, Table **S2**. The levels were lower in all measured ONPs than in the two snus products. The per-pouch ash level in ONPs was, on average, 76% lower (range, 47 to 88%) as compared with the reference snus and 74% lower (range, 41 to 86%) relative to the commercial snus product; on a per-gram basis, the ONPs contained 66% less ash than both the reference and commercial snus product (range, 30 to 83% lower than reference snus; 31 to 83% lower than commercial snus).

#### Elements

Seven ONPs and both snus products had quantifiable amounts of arsenic. Arsenic contents of the other ONPs were not quantifiable. Per pouch, the commercial ONPs had, on average, 94% lower (range, 87 to 98%) arsenic content than the reference snus product, and 91% lower (range, 82 to 97%) arsenic content than the commercial snus product. Per gram, the commercial ONPs contained 90% and 92% less arsenic relative to the commercial and reference snus samples, respectively.

Two commercial ONPs, the unflavoured zero-nicotine ONP and both snus samples had quantifiable beryllium contents, while the other nine ONPs had content too low to quantify. The commercial ONPs had on average, 93% lower (range, 86 to 97%) beryllium content than the reference snus product, and 94% lower (range, 87 to 97%) content than the commercial snus product. On a per-gram basis, the ONPs contained 91% and 93% less beryllium relative to the reference and commercial snus products, respectively.

Both snus samples had quantifiable cadmium content, but values for all ONPs were below the LOQ. The ONPs had on average, 99% lower cadmium content as compared with the two snus products on both a per-pouch and per-gram basis.

None of the ONPs contained quantifiable chromium content, but both snus products had quantifiable levels. The ONPs had on average, 87% lower (range, 85 to 93%) chromium content than the reference snus product and 84% lower (range, 83 to 91%) content than the commercial snus product per pouch. On a per-gram basis, the ONPs had an average chromium content that was 80% and 81% lower relative to the commercial and reference snus products, respectively.

One ONP (CP2) and both snus products had quantifiable lead content, while the other ONPs had values < LOQ. Per pouch, the ONPs had 90% lower (range, 79 to 95%) lead content than the reference snus product, and 87% lower (range, 73 to 94%) content than the commercial snus product. On a per-gram basis, the ONPs had a mean lead content that was 84% and 86% lower relative to the commercial and reference snus products, respectively.

Mercury was quantifiable in both snus samples, but not in any of the ONPs. Per pouch, the ONPs had 97% lower (range, 93 to 97%) mercury content than the reference snus product, and 95% lower (range, 91 to 96%) content than the commercial snus product. On a per-gram basis, the mercury content was 94% lower in ONPs than in both the snus products.

Nickel was found at quantifiable levels in five ONPs and both snus samples; the other ONPs had a nickel content < LOQ. On average, the ONPs had 96% lower (range, 89 to 99% lower) nickel content than the reference snus product, and 95% lower (range, 86 to 98% lower) content than the commercial snus product. On a per-gram basis, the nickel content of ONPs was 93% and 94% lower than that of the commercial and reference snus products, respectively.

All ONPs and the commercial snus product had selenium content too low to quantify; however, the reference snus product had a quantifiable selenium level. Relative to the reference snus product, the ONPs had 43% lower selenium (range, 37 to 69%) on a per-pouch basis. On a per-gram basis, the selenium content was 18% lower in the ONPs than in the reference snus product.

Sodium was quantifiable in all samples. One ONP (Velo Salt Freeze, 20 mg) had higher sodium per pouch, and another ONP (CP1) had higher per-gram sodium levels than both snus products. The sodium contents of the other ONPs were lower than those of the snus samples. On average, the ONPs had 48% lower (range, 16% higher to 72% lower) sodium content than the commercial snus product, and 49% lower (range, 14% higher to 73% lower) content than the reference snus product. On a per-gram basis, the commercial ONPs had 27% and 34% lower sodium content than the reference and commercial snus products, respectively.

#### Radioisotopes

Of the 27 radioisotopes analysed, only three (^14^C, ^210^Po and ^40^K) were quantified. Method blank samples were not quantifiable for any measured isotope.

Carbon-14 was not detected in either snus product but was quantified in all 11 commercial ONPs. It should be considered that the LODs for the snus samples were up to 6-fold higher than those for the ONPs. As several ONPs had quantifiable ^14^C contents below the snus LODs, it is not possible to accurately estimate the overall % differences between snus products and ONPs. However, two ONPs had ^14^C contents higher than the LOD for the reference snus product and three were higher than the LOD for the commercial snus product. The mean value for these five observations was 37% higher in these ONPs than either snus product.

Polonium-210 was quantified in both snus products and in one ONP (Velo Hybrid Tropic Breeze 6 mg). On a per-pouch basis, the ^210^Po content of the ONPs was, on average, 78% lower (range, 56 to 94%) than the reference snus product, and 83% lower (range, 65 to 95%) than the commercial snus product. On a per-gram basis, the ONPs had a mean ^210^Po content that was 69% and 78% lower than that of the reference and commercial snus products, respectively.

Potassium-40 was quantified in both snus products but no ONP samples. On average, the mean per-pouch ^40^K content of the ONPs was estimated to be 89% lower (range, 87 to 93%) than that of the reference snus product and 91% lower (range, 89 to 94%) than that of the commercial snus product. On a per-gram basis, ^40^K content was 83% and 88% lower in the ONPs than in the reference and commercial snus products, respectively.

### Comparative toxicant contents of ONPs and snus

To summarise the key findings, we compared the mean percentage differences between the commercial ONPs and the two snus products (Supplementary File 2, Table S7 and Fig. 2). Across all samples, the mean content of 11 measurands was higher in ONPs than in either snus product while that of 51 measurands was lower in the ONPs. The measurands present at higher levels were pH, fluorene, both 1 and 2-methylnaphthalene, acrolein, total alkaloids, nicotine, glycerol, *N*’-nitrosodibenzylamine, ethyl carbamate and ^14^C. The presence of ^14^C relates to those two to three ONPs with quantified values higher than the reference and commercial snus products LODs, respectively. The measurands present at the highest relative levels were glycerol (1 ONP), *N*-nitrosodibenzylamine (1 ONP) and ethyl carbamate (3 ONPs).

The measurands present at lower levels in ONPs than in snus included members of all chemical classes of quantified compounds from this study. Of the 51 lower level measurands, 27 were > 90% lower in ONPs than in the snus products.

The ONP chemical contents measured in this study were compared with the GOTHIATEK^®^ standard [57]. Swedish Match developed this voluntary quality standard to monitor the levels of 16 undesired compound groups in manufactured snus. The identities of the GOTHIATEK^®^ compounds, their corresponding limit values, and the magnitude of these compounds in the ONPs and snus products measured in the present study are provided in Supplementary File 2, Table S5. Although most GOTHIATEK^®^ constituents were measured in the present study, three measurands were not: nitrite, ochratoxin A and the agrochemicals. However, Supplementary File 2, Table S5 shows that all products complied with the GOTHIATEK^®^ standard for the compounds measured in the present study. The contents of most analytes in the study ONPs were substantially lower than the GOTHIATEK^®^ standard limits, shown in Figs. 3 and 4.

To focus further on compounds that might play a key role in THR, in Table 4 we determined the percentage differences between the two product categories for the 18 priority toxicants mandated or recommended for lowering and high priority for disclosure and monitoring in cigarette smoke by WHO TobReg [56]. Although quantitative data is not available for all of the 18 chemicals, it is notable that ONPs contained, on average, significantly lower levels of acetaldehyde, formaldehyde, cadmium, NNK and NNN as compared with either snus product. In contrast, acrolein levels were elevated in the two ONPs in which it was quantifiable.

### Comparison of toxicant contents with cigarette smoke

To assess the potential role of ONPs in THR further, we compared our findings with those from a previous analysis of the emissions from a reference cigarette [17], as shown in Supplementary File 2, Table S6. Although that study was comparable in size (126 measurands) to the current analysis of ONPs (132 measurands), only 58 analytes were common to both studies (Fig. 5 and Supplementary File 2, Table S6), largely due to the physical differences between aerosol and solid matrices.

Among the 58 common analytes, 10 were < LOQ for both 1R6F reference cigarette and all ONP samples, and were excluded from further analysis, as per the data analysis protocol. Of the remaining 48, eight were higher in ONPs than in 1R6F reference cigarette emissions (nicotine, glycerol, propylene glycol, anabasine, N’-nitrosodiethanolamine, nicotine-1’-oxide, nickel and ethyl carbamate). Nicotine-1’-oxide was the measurand present at the highest levels in ONPs relative to cigarette smoke. Among the 40 compounds lower in ONPs than in cigarette smoke, 34 were > 90% lower in ONPs.

We also compared 1R6F reference cigarette smoke and snus (Supplementary File 2, Table S6 and Fig. 6). Eight measurands were < LOQ for both 1R6F reference cigarette and snus samples and were excluded from further analysis. Of the remaining 50 measurands, overall, 18 compounds were higher in the snus products than in 1R6F reference cigarette smoke; of these, nicotine − 1’-oxide, chromium and nickel were at the highest relative levels in the snus products. Among the 32 compounds lower in snus than in 1R6F reference cigarette smoke, 22 were > 90% lower.

Considering the WHO TobReg9 toxicants [56], carbon monoxide was not analysed as it is a permanent gas and unlikely to be entrained in either snus products or ONPs to any significant degree. The remaining measured eight toxicants (prioritised for lowering) were 98.7 to 99.9% lower in the ONPs than in cigarette smoke. Five of the nine toxicants proposed for disclosure and monitoring [56] measured in this study were 97.7 to 99.9% lower in ONPs than in cigarette smoke. The snus products showed a similar trend to ONPs for the nine toxicants, except that the levels of NNK were not as reduced relative to 1R6F reference cigarette smoke to the same extent that they were for ONPs, with NNN having 28% higher levels compared to 1R6F cigarette smoke. Regarding the toxicant levels of four of the five measured compounds were lower in the snus products than in 1R6F reference cigarette smoke; however, cadmium levels were higher in the snus products than in 1R6F reference cigarette smoke.

## Discussion

Herein, we have reported a broad yet detailed examination of the toxicant contents of a commercial Swedish snus product, a reference snus product (1S4), and 11 commercially available ONPs. We have presented data on a wide range of organic and inorganic toxicants, comparing the magnitude of each across the two product categories. We have also compared their contents to that of smoke from a reference cigarette (1R6F).

### Comparative chemical complexity of ONPs and snus

Of the 132 measurands investigated in this study, 70 were not present at quantifiable levels in any of the ONPs or snus products, while 62 were quantified in one or more products. Compound classes that were commonly identified at quantifiable levels in at least one of the study products included PAHs (18/21 analytes), carbonyls (3/4), nicotine-related alkaloids (9/9), TSNAs (4/7), and elements (8/8).

Of the 62 quantified measurands, 58 were quantifiable in at least one snus sample, whereas 47 were quantified in at least one of the ONPs. The following 15 measurands were quantifiable in one or both snus samples, but not in any of the ONPs: benzo[j]fluoranthene, nitrate, total bacterial count and mould count, four TSNAs, 3-(methylnitrosamino) propionic acid, *N*-nitroso-l-proline, Cadmium, Chromium, Mercury, Selenium and Potassium-40. By contrast, only four measurands were quantifiable in one or more ONPs but not in either snus: acrolein (2/11), glycerol (1/11), NDBzA (1/11), and the radioisotope ^14^C (11/11).

The reasonably comparable numbers of analytes quantified in ONPs and snus products may seem surprising because the tobacco contained in snus is likely to be much more compositionally complex [49] than the physical substrate and combination of ingredients used in ONPs. However, the present study’s aim was not to completely characterise the chemical composition of these two oral nicotine products (ONPs and snus), but rather to focus on specific measurands, including toxicants. On this basis, there seems to be broadly similar numbers of toxicants present at quantifiable levels in the snus products and ONPs.

### Levels and potential sources of compounds identified in ONPs and snus

The current chemical analysis of ONPs investigated a wider range of compounds than any previous study. While sources of and precursors to many of these compounds in snus products have been discussed previously [21, 41–46], understanding the sources of compounds identified in ONPs is a less developed area.

To support the discussion below, we have collated previously reported chemical content data on ONPs from the published literature and summarised it in Table 5.

#### Measurands quantified in both ONPs and snus

Although the snus products and ONPs are physically similar, on average, the 11 ONPs were slightly lighter, slightly drier and more alkaline than the two snus comparators. These differences are a consequence of product design and formulation.

A total of 18 of the 21 PAHs (all, except for benzo[a]pyrene, benzo[e]pyrene, and perylene) were quantified in ONPs and/or snus. Back et al. [33] previously analysed ONPs for seven PAHs, but did not detect any at levels > LOQ. Because they used a less-sensitive analytical method (substantially higher LOQs), their results cannot be directly compared with our own. Consistent with the present findings, PAHs have been commonly found in trace amounts in STPs including snus [41].

Comparable (± 30%) contents of five PAHs (acenaphthene, acenaphthylene, fluorene, naphthalene and 1-methyl naphthalene) were found in ONPs and snus products, while higher levels of 2-methylnaphthalene were found in some ONPs (Supplementary File 2, Table S7). These six PAHs are two- or three-membered ring compounds, with carbon numbers of C_10_ to C_12_. In contrast, levels of 12 chemically larger PAHs (≥ C_13_, possessing three or more aromatic rings) were substantially lower in ONPs than in snus.

The reason for this differentiation in the content of C_10_–C_12_ and ≥C_13_ PAHs is unclear but is likely to be a consequence of their formation mechanisms. To explore these differences, we applied the source apportionment approach previously used for STPs [41]; however, the calculations simply confirmed the trend of different PAH ratios between the ONP and snus samples, suggesting different source mechanisms. Previously, PAHs in snus were attributed to contamination from petrogenic and wood/biomass combustion, most likely acquired via environmental pollution pathways [41]. The trace levels of PAHs in ONPs appear to arise from different sources than snus, potentially during production of the pouch and substrate materials, which originate from cellulosic materials, and this area warrants further exploration in future studies.

All nicotine-related compounds analysed were quantifiable in both snus samples, but not in all ONPs. Nicotine, nornicotine, and nicotine-1-oxide were present in all 11 ONPs. The other nicotine-related compounds were quantifiable in fewer ONPs. These observations are generally consistent with a previous study that used analytical methods with comparable LODs and LOQs [30]. Here, nicotine was generally present at higher levels per pouch, whereas the nicotine-related compounds were present at lower levels (65.7 to 98.5% less) in ONPs than in the snus products (Supplementary File 2, Table S7). In the two snus samples, expressed as the sum of these nicotine-related compounds, nicotine on average accounted for 94% of the total, and anatabine, nornicotine and nicotine − 1-oxide accounted for 1 to 3%. However, in ONPs the average contribution of nicotine to the total of nicotine-related compounds was 99.1%, and nicotine − 1-oxide contributed 0.7% of the total.

Tobacco is the reported source of the nicotine-related alkaloids [30]. Nicotine in ONPs is often (but not exclusively) tobacco-derived [58] and is purified to stringent pharmaceutical standards [59]. Nevertheless, alkaloids, such as nornicotine, anatabine and anabasine, are present as minor impurities in pharmaceutical nicotine [30]. Compounds such as cotinine, nicotine-*N*’-oxide, myosmine and β-nicotyrine are regarded as degradation products formed through microbial action, oxidation or exposure of nicotine to elevated temperatures. In summary, their presence in ONPs is probably a consequence of the use of tobacco-derived nicotine in these products and its subsequent reactions. However, the lower levels in ONPs relative to snus suggest that the purification process used to produce pharmaceutical nicotine is partially effective at removing these compounds, while an alternative suggestion is that the ageing effect could also be considered as having an impact.

Unexpectedly, several nicotine-related impurities and degradation products (but not nicotine or total nicotine alkaloids) were quantified in the nicotine-free pouch. The levels were higher in this sample than in some ONPs with higher nicotine content and were also higher than previously reported [30]. As described above, in nicotine-containing ONPs these compounds arise from nicotine and its sources; their presence in a nicotine-free sample is therefore challenging to explain. Similar levels were measured for each of the 7 compounds, which differs markedly from the relative quantities found in the nicotine-containing ONPs. The reasons for these observations are unclear, but they are consistent with the possibility of an analysis error.

The detection of NDELA in all study products, and its quantification in the two snus products and six ONPs (at 16 to 76% lower levels than snus, ONPs were quantified between 2.8 and 3.8 ng per pouch) is surprising as, historically, NDELA was found in tobacco due to application of the now-banned sucker growth inhibitor maleic hydrazide diethanolamine salt to crops [60]. NDELA is biologically active, causing liver tumours in rats [61, 62], and therefore a compound of concern for ONPs where present. NDELA is formed by nitrosation of diethanolamine and triethanolamine, which are widely used across many industrial applications and in the production of a wide range of consumer products, such as paper, printing inks and cosmetics. It’s detection in this study may reflect contamination or trace impurities in some materials used in these products. Further studies are needed to understand its presence in contemporary snus products and ONPs.

Acrylamide was quantified in five ONPs and both snus samples. No other studies have reported acrylamide in ONPs (Table 5); however, acrylamide was detected in all of the 32 snus products previously analysed and was quantifiable (74 to 276 ng/g) in 31 of those samples [43], consistent with the content of the snus products in the present study (142 to191 ng/pouch). The presence of acrylamide in the snus products is thought to be the consequence of base-mediated reactions of asparagine (in tobacco) during manufacture [43]. The observation of acrylamide in five (alkaline) ONPs in the present study might therefore indicate that asparagine is in, or has been in contact with, these samples; alternatively, acrylamide is widely used industrially in sectors such as in textile, paper and pulp manufacturing and water treatment. These may represent a possible route to acrylamide contamination of ONPs.

Ethyl carbamate was quantified in three ONPs at 35 to 67 ng per pouch and in one snus at 13.5 ng per pouch. In contrast, quantifiable levels of ethyl carbamate were not previously found in two ONPs [33]. In an earlier study of snus chemistry, ethyl carbamate was quantified in 19 of 32 Swedish snus samples at a mean of 35 ng/g (close to the LOQ in the present study). Its presence was attributed to reactions of residual ethanol used as a flavour application process aid in some snus products [46], it has also been noted to be a process contaminant that can form as a byproduct during fermentation [63]. Ethyl carbamate is also used as an intermediate in resin and plastic manufacturing, which might potentially be a source of its presence in the three ONPs of the current study.

Acetaldehyde and formaldehyde were quantified in all study products, consistent with previous studies on ONPs and snus products (Table 5). Levels of acetaldehyde in ONPs were substantially lower (99%), while formaldehyde levels were slightly lower (22%) than in the reference snus product (Supplementary File 2, Table S3). Among the ONPs, there was no obvious relationship between the content of either carbonyl or nicotine strength or flavour. Coleman and Perfetti [64] proposed that tobacco carbonyls are formed by thermal reactions of amino acids such as glycine (formaldehyde) and α-alanine (acetaldehyde) via Strecker degradation reactions. Such a mechanism may explain the presence of these carbonyls in our study snus samples, but it is not compatible with the production and composition of ONPs where amino acids are not a standard component. The ratio of acetaldehyde to formaldehyde differed considerably between snus products (median, 6.53; 6.16 and 6.91) and ONPs (median, 0.19; 0.07 and 0.31), suggesting that the source of these carbonyls is likely different between these product categories. Acetaldehyde is a contaminant found in plastics, varnishes and resins, and food flavourings; formaldehyde is a contaminant in textiles, and paper manufacturing. Off-gassing from these sources in manufactured and packaged ONPs may potentially explain their presence in ONPs.

All measured study products, contained quantifiable levels of sodium, albeit at much lower levels in the ONPs than the snus products. This is consistent with salt being a common ingredient in both snus products and ONPs. All study products also contained ash (residual inorganic content), but levels were lower in ONPs, suggesting a higher organic content than in the snus products. Both snus product samples contained quantifiable levels of ^210^Po and all non-radioisotope elements analysed other than selenium (quantifiable in one). In contrast, ^210^Po was quantified in only one ONP, at a substantially lower level (1.262 mBq). Quantifiable ^210^Po was not previously found in two ONPs [33] using a less sensitive analytical method (LOQ, 5 mBq). ^210^Po is a naturally occurring radionuclide that accumulates in leafy plant material following decay of atmospheric radon. It could, in principle, possibly be present in the ONP as a trace contaminant in the cellulosic materials used in its manufacture.

Arsenic, beryllium, lead and nickel were quantifiable in a few ONPs. The levels of arsenic, nickel and lead are consistent with previous findings in ONPs (Table 5). Although quantifiable beryllium levels were not previously found, the lowest LOQ of the analytical method was 2.5 ng per pouch. Regarding snus, trace elements are incorporated into tobacco during cultivation, mainly from soil and atmospheric sources [37]. In ONPs, by contrast, these elements most likely derive from impurities in the ingredients or from the manufacturing equipment.

With oral STPs, many elements have limited solubility in saliva [65, 66], resulting in limited extraction from oral STPs, such as snus, during use. Rostron et al. [67] analysed biomarker concentrations in the NHANES database. They found that blood cadmium, blood mercury and urine arsenic levels were lower, but not significantly so for STP users compared to non-tobacco users, while blood lead levels were significantly higher by about 30%. Collectively, these data suggest very little uptake of arsenic, mercury or cadmium from STPs during use. It is plausible that similar processes may occur during ONP use. However, given the likely differences in mechanisms for incorporation in ONPs vs. snus products, and possible resulting differences in elemental speciation, further work is required to examine their extractability from ONPs during use.

#### Compounds quantified in ONPs but not snus products

Acrolein was not detected in nine of the ONPs in this study but quantified in two ONPs (0.12 and 0.28 µg/pouch) and detected but not quantified in the reference snus product (1S4). Only one other study has examined acrolein in ONPs, but the levels were not above the reported LOQ (0.05 µg/pouch) (Table 5). Similarly, in a study of 32 STPs, one portion snus contained acrolein at 0.337 µg/g, but the compound was too low to quantify in the other 31 samples [36]. Given that the International Agency for Research on Cancer (IARC) has classified acrolein as probably carcinogenic to humans (Group 2 A) [68], the origin of the volatile aldehydes is important from a health perspective. In cigarette smoke, aldehydes mostly derive from the pyrolysis of tobacco carbohydrates [69] and, particularly for acrolein, from the degradation of glycerol. Notably, one of the two ONPs with quantifiable acrolein was the only sample with quantified levels of glycerol (commonly added to nicotine products as a humectant); thus, if used, glycerol may possibly be a source of acrolein in ONPs. Acrolein could also, in principle, be present due to added flavour compounds (whether as contaminants or reaction products) or as contaminants in the ONP substrate and/or pouch material. Further research in this area is required to test these possibilities.

Among the study products, NDBzA was quantified in one ONP at 3.34 ng/pouch. No previous studies have analysed ONPs for this nitrosamine; however, NDBzA was detected in two snus samples of 70 STPs analysed [38] and, to our knowledge, that is the only study to have reported the compound in any tobacco product. Formation of the analogous dialkyl nitrosamines (such as NDMA) in tobacco is explained by the nitrosation of dialkyl amines. Here, the presence of NDBzA in a single sample may indicate that dibenzyl amine was in, or had come in contact with, the ONP.

Lastly, the radionuclide ^14^C was quantified in all the measured 11 ONPs (6 to 24.9 mBq), but in neither snus sample (which had relatively high LODs of 15 to 19 mBq). ^14^C has not been examined previously in ONPs, but it was previously quantified in 31 of 32 snus samples (135 to 435 mBq) [44]. The relatively high LODs may obscure the presence of lower levels of ^14^C in the snus samples. Some radionuclides, including the ß-emitter ^14^C, are continuously produced in the earth’s atmosphere by cosmic ray bombardment [44] and entrained in natural carbon sources during plant growth. Its presence in all 11 ONPs may reflect a higher carbon content of ONPs relative to snus, consistent with the ash data reported here.

#### Compounds quantified in snus but not in ONPs

Our finding of undetectable levels of NAB, NAT, NNK, NNN in any of the study ONPs is consistent with five previous ONP studies; however, a recent study using more sensitive analytical methodology quantified TSNAs in 6 to 24 ONPs [34] (Table 5). While the LOQs in the latter study were slightly more sensitive than those of the current study, the levels of TSNAs reported by Mallock et al. [34] would have been quantifiable by our methodology.

TSNAs are commonly found in tobacco due to the nitrosation of tobacco alkaloids, often catalysed by nitrate reductase enzymes. For example, the four TSNAs were previously quantified in all of the 70 analysed STPs [38]. Their presence at very low levels in ONPs may be due to trace impurities in pharmaceutical nicotine. In support of this hypothesis, TSNAs have been found in commercially available samples of purified nicotine at levels of 0.5 to 50 ng/g [70]. Assuming these TSNAs were present at similar levels in the nicotine in our ONPs, they would be below the detection limit of the analytical method that we used. In short, it is possible that the ONPs that we analysed may contain TSNAs, but at currently undetectable trace levels.

The nitrosacids MNPA and NPRO were not measurable in any of the ONPs but were quantified in both snus products, consistent with a recent study of 76 STPs [39]. Precursors to nitrosacids are the corresponding amino acid and nitrosating agents. The absence of quantifiable nitrosacids in our study ONPs suggests a lack of the respective amino acid precursors and/or a comparatively unfavourable nitrosation environment in ONPs compared to snus products.

Total bacterial and mould counts, as well as nitrate, were measurable in the snus samples (total mould and bacterial count only above the RL in the reference snus product) but not in the ONPs, (Supplementary File 2, Table S3). As a typical agricultural product, tobacco has a rich and diverse bacterial and mould population. Snus tobacco is pasteurised at ~ 100 °C for several hours during production, limiting the development of microbiological counts as compared with other STPs [9]. The absence of quantifiable levels of total bacterial and mould count and nitrate in the present study is consistent with the identity of ONPs as tobacco-less products manufactured under conditions unfavourable for the development of microbiological species and is also consistent with the very low TSNA levels in the ONPs.

Benzo[j]fluoranthene was quantified in both snus samples but was not quantified in the 11 ONPs (not detected in 10 ONPs). No other studies have analysed ONPs for this PAH (Table 5), but our finding is consistent with our above comments concerning the absence of larger PAHs (> C_13_) in ONPs. The levels of benzo[j]fluoranthene in snus measured here are approximately one-third of the average reported for 32 snus samples from samples produced in 2008 [41]. It is possible that PAH levels have fallen over time due to improved manufacturing practices.

Cadmium was quantified in both snus products (216 to 219 ng per pouch and 228 to 255 ng per gram), but not in any of the ONPs, in keeping with five previous studies that examined the elemental composition of ONPs (Table 5). Cadmium enters plants, including the tobacco plant, primarily through the roots and is then transported rapidly to the leaf tips. The amount taken up by the roots is dependent on the soil concentration and pH. IARC has categorised cadmium as a Group 1 carcinogen (carcinogenic to humans) [71], but it is commonly found in food, and the European Food Safety Authority Panel (EFSA) has established a tolerable weekly intake for cadmium in foodstuffs of 2.5 µg/kg bw per week [72] which both snus products are noted to be significantly below.

Similarly, chromium, mercury and selenium were quantified in at least one of the snus products, but not in the ONPs included in this study. Some studies have reported quantifiable levels of chromium in ONPs, but previous findings for mercury and selenium are consistent with ours (Table 5). Lastly, McAdam et al. [44] reported that the greatest contribution to the overall radioactivity measured in tobacco was from the β-emitter ^40^K; the same conclusion can be drawn from the two snus products analysed in this study. ^40^K accounts for 0.012% of all naturally occurring potassium and is entrained into tobacco during cultivation. Its absence at detectable levels in ONPs suggests the absence of any form of potassium at a significant level in these products.

#### Compounds not detected or not quantified in either ONPs or snus

Benzene, 1,3-butadiene and isoprene were not detected in any of the study products. Of these three compounds, only benzene has been previously analysed in ONPs (Table 5) and was also not detected. These three volatile compounds have relatively high vapour pressures under ambient conditions; thus, it is unlikely that they would be entrained in ONPs under normal environmental conditions. In keeping with five previous studies (Table 5), benzo[a]pyrene, benzo[e]pyrene and perylene were not detected or not quantified in our study products.

Three of the TSNAs (NNA, NNAL, iso-NNAL) were detected in every study product but not quantified. NNA and iso-NNAL are generated in a common nitrosation pathway of nicotine. In contrast, NNAL is generated in a different nitrosation pathway as a daughter product of NNK (itself a nitrosation product of nicotine).

Other than NDBzA and NDELA, neither the snus products nor ONPs had quantifiable amounts of volatile and involatile nitrosamines. Most of these compounds were not detected, although in some samples NDiPLA (5 ONPs) and NPIP (8 ONPs, 2 snus) were detected but not quantified. NDiPLA can form in industrial raw materials, and NPIP can form in water systems and can be generated in rubber manufacturing. Apart from MNPA and NPRO in the snus samples, the nitrosacids were not measurable (< RL) in the present study.

The four aromatic amines and acrylonitrile were not detected in any of the ONPs, however two of the aromatic amines were detected but not quantified in the commercial snus and one in the snus reference product (1S4). Hydrazine was detected (but not quantified) in only one study product, an ONP. Given the volatility of this compound, this finding is unexpected and its source in the ONP is as yet unexplained.

Coumarin and the two angelica lactones were not detected in any of the study products. One other study of coumarin in ONPs reported it as not quantifiable (Table 5). Regarding STPs, coumarin was previously detected in only 3 of 33 snus, and α-angelica lactone was quantified in 3 of 30 Swedish snus, while β-angelica lactone was tentatively identified in 1 sample [45]. Coumarin and angelica lactones have been reported to be natural tobacco constituents, as well as flavouring components in various products [45]; their absence from ONPs is therefore not surprising.

We did not detect crotonaldehyde in any of the ONPs, consistent with five other studies of these products (Table 5). Crotonaldehyde was present at non-quantifiable levels in the commercial snus product, which is again consistent with a previous study in which it was detected but not quantified in 1 of 32 snus samples [36].

Aflatoxins were below RL in both snus products and all the ONPs in this study; this is consistent with recent studies on these compounds in different ONPs (Table 5). As these compounds are the products of microbiological species, their absence from ONPs is plausible given the lack of quantifiable microbiological measurands in these products.

Similarly, our non-quantifiable levels of total and reducing sugars in all the study products is consistent with previous analyses of these compounds in snus [21]. We would not expect to find them in ONPs unless specifically added as an ingredient in the product formulations.

There has been one other investigation of radionuclides in ONPs, which similarly reported non-quantifiable levels of ^210^Po, ^235^U and ^238^U [33]. Because ONPs are produced from heavily processed materials rather than from natural biomass (such as tobacco), it is possible that the processing steps will result in low levels of radionuclides.

### Quantitative comparison of ONP and snus contents

Overall, 11 measurands were higher in the ONPs than in the two snus products included in this study, but 51 were lower in ONPs than in snus (Supplementary File 2, Table S7 and Fig. 2). It should be noted that carbon-14 has been included in the list of compounds with a higher presence in the ONPs than snus due to the uncertainty of the carbon-14 content of snus arising from comparatively higher LODs compared with the ONPs as well as observations that two to three of the 11 ONPs measured had contents higher than the reference and commercial snus products LODs.

Notwithstanding, of those 51 compounds lower in ONPs than snus, 27 were substantially lower (> 90%) in the ONPs. Regarding the 18 key cigarette smoke compounds identified by WHO TobReg, levels of acetaldehyde, formaldehyde, cadmium, NNK and NNN were much lower in all ONPs; however, acrolein was higher in two ONP samples.

Regarding the GOTHIATEK^®^ standard, all of the study products met the limit values for undesired compounds stipulated by Swedish Match [57]. The ONPs level of many GOTHIATEK^®^ compounds (NNN, NNK, acetaldehyde, arsenic, cadmium, chromium, lead, mercury and nickel) were substantially lower than the corresponding snus levels. The other measured GOTHIATEK^®^ compounds were either below LOQ or not detected for both the ONP and snus samples.

This study did not measure some GOTHIATEK^®^ compounds (agrochemicals, nitrite and ochratoxin A). Agrochemicals are likely to be of limited relevance to ONPs, due to their non-agricultural composition. Nitrite was not measured, but nitrate, microbiological activity and TSNAs contents of ONPs were either below LOQ or below RL, and substantially lower than levels in the study snus products. In four previous studies of ONPs (Table 5) nitrite has not been present at quantifiable levels in ONPs. Similarly, although ochratoxin A was not measured, the mycotoxins measured in this study (aflatoxins) and mould, bacterial and yeast counts were below the method RL, and largely lower than those in snus. Azzopardi et al. [20] also did not find either aflatoxins or ochratoxin A above the method RL with similar ONPs. Hence, the data for ONPs shown in Supplementary Table File 2, S5 suggests that the study ONPs are compatible with the GOTHIATEK^®^ standard chemical limits.

### Toxicant contents of ONPs and snus compared to cigarette smoke

Based on the data of Forster et al. [17], 18 measurands were higher in the snus products than in the reference cigarette smoke (1R6F), including seven nicotine-related compounds, seven metals, propylene glycol, ethyl carbamate and two nitrosamines (Supplementary File 2, Table S6 and Fig. 6). However, 32 measurands were substantially lower in snus than in the reference cigarette smoke (1R6F). These observations are consistent with the findings of recent FDA literature reviews (13, 73), which concluded that exclusive use of snus which meets the GOTHIATEK^®^ standard is associated with significantly lower risks of oral cancer, lung cancer, myocardial infarction, chronic obstructive pulmonary disease and stroke than cigarette smoking.

The differences between ONPs and the reference cigarette smoke (1R6F) were even more marked, with only eight measurands higher in ONPs than in smoke (Table S6 and Fig. 5). Of these compounds, three were ingredients (nicotine, glycerol and propylene glycol) and two were nicotine impurities; there were also a metal, a nitrosamine and ethyl carbamate. Notably, all these compounds were also higher in snus than in cigarette smoke, except glycerol. In all, 40 of the 48 measurands, covering all chemical classes, were substantially lower in ONPs than in cigarette smoke.

Overall, 13 of the 17 measured WHO TobReg toxicants were 97.7 to 99.9% lower in ONPs than in the reference cigarette smoke (1R6F). Furthermore, levels were 98.7 to 99.9% lower in ONPs than in the reference cigarette smoke (1R6F) for the nine toxicants prioritised for mandated lowering, and 97.7 to 99.9% lower for the toxicants proposed for disclosure and monitoring, where measured. The comparison of the snus products to the reference cigarette smoke (1R6F) was similar, albeit with less substantial reductions (63 to 99.9%) for some of the compounds proposed for mandated reductions. Of the WHO TobReg compounds proposed for mandated lowering and disclosure and monitoring, the snus products had increased cadmium and NNN content relative to the reference cigarette smoke (1R6F).

**Table 1:** The 14 study products

Product	Manufacturer	Flavour^1^	Nicotine (mg/pouch)
Oral nicotine pouch			
*BAT Products*			
Non-commercial product			
Unflavoured 0 mg	Nicoventures Trading Ltd	None	0
Commercial products			
Velo Hybrid Tropic Breeze 6 mg	Nicoventures Trading Ltd	Complex	6
Velo Hybrid Urban Vibe 10.9 mg	Nicoventures Trading Ltd	Complex	10.9
Velo Salt Swedish mint 6 mg	Nicoventures Trading Ltd	Simpler	6
Velo Salt Freeze 20 mg	Nicoventures Trading Ltd	Simpler	20
Velo Sweet Tropic Breeze 6 mg	Nicoventures Trading Ltd	Complex	6
Velo Sweet Tropic Breeze 10.9 mg	Nicoventures Trading Ltd	Complex	10.9
Velo Salt Ice Cool 10 mg	Nicoventures Trading Ltd	Simpler	10
Velo Sweet Polar Mint 6 mg	Nicoventures Trading Ltd	Simpler	6
Velo Sweet Polar Mint 10 mg	Nicoventures Trading Ltd	Simpler	10
*Competitor Products*			
CP1 – Competitor Product 1 (Mini, Mint)			8
CP2 – Competitor Product 2 (Slim Spearmint)			9
Snus			
1S4 reference	CTRP	N/A	7.3
General Classic Blend White Portion Large Snus	Swedish Match	N/A	7.2

CTRP, Center for Tobacco Reference Products (University of Kentucky); N/A, not applicable

^1^ Simpler flavours are defined as having a lower number of ingredients or natural oil inclusion compared to flavours defined as Complex

**Table 2:** Summary of methods, replicates and validation status for the analytes examined in this study

Analyte group	Measured by	No. of replicates	Analysis method	Analytical method validation status	
				Snus	ONPs
1. Acrylamide	Eurofins CRL, USA	3	Extraction, LC-MS/MS	Pre-validated	Validated in study
2. Aflatoxins	Eurofins FFT, Sweden	3	Extraction, HPLC	Pre-validated	Pre-validated
3. Alkaloids (total)	Eurofins CRL, USA	3	Extraction, CFA colorimetric	Pre-validated	Pre-validated
4. Aromatic amines	Labstat Int.	3	Extraction, GC-MS	Pre-validated	Validated in study
5. Angelica lactones	External laboratory, UK	3	Extraction, GC-MS	New method	New method
6. Ash	Eurofins FFT, Sweden	3	Combustion, gravimetry	Pre-validated	Pre-validated
7. Carbonyls	Labstat Int.	3	Extraction, GC-MS	Pre-validated	Pre-validated
8. Coumarin	Labstat Int.	3	Extraction, LC-MS/MS	Pre-validated	Validated in study
9. Ethyl carbamate	Labstat Int.	3	Extraction, GC-MS	Pre-validated	Validated in study
10. Humectants	Labstat Int.	3	Extraction, GC	Pre-validated	Validated in study
11. Hydrazine	External laboratory, UK	3	Headspace, GC-MS	New method	New method
12. Mass	All	3	Gravimetry	Pre-validated	Pre-validated
13. Metals and metalloids	Eurofins CRL, USA	3	Acid digestion, ICPMS (RC)	Pre-validated	Pre-validated
14. Mercury	Eurofins CRL, USA	3	Thermal digestion, AAS	Pre-validated	Pre-validated
15. Microbiological counts	Eurofins CRL, USA	3	Plate counting	Pre-validated	Pre-validated
16. Nicotine	Eurofins CRL, USA	3	Extraction, GC-FID	Pre-validated	Pre-validated
17. Nicotine-related compounds	Labstat Int.	3	Extraction, LC-MS/MS	Pre-validated	Validated in study
18. Nitrate nitrogen	Eurofins CRL, USA	3	Extraction, CFA/ colorimetric	Pre-validated	Pre-validated
19. Nitrosamines (TSNAs)	Labstat Int.	3	Extraction, LC-MS/MS	Pre-validated	Pre-validated
20. Nitrosamines (NNA, NNAL, iso-NNAL)	External laboratory, UK	3	Extraction, LC-MS/MS	New method	New method
21. Nitrosacids	External laboratory, UK	3	Extraction, LC-MS/MS	New method	New method
22. Nitrosoalkanol-amines and volatile nitrosamines	Labstat Int.	3	Extraction, LC-MS/MS	Pre-validated	Validated in study
23. N-Nitroso-benzylamine	Labstat Int.	3	Extraction, LC-MS/MS	Pre-validated	Validated in study
24. Polycyclic aromatic hydrocarbons (PAHs)	Labstat Int.	3	Extraction, GC-MS	Pre-validated	Pre-validated
25. pH	Eurofins CRL, USA	3	Extraction, glass electrode	Pre-validated	Pre-validated
26. Radionuclides	External laboratory, UK	1 composite gross sample	Standard radiological methods	Validated for plant/vegetable matrices	
27. Sodium ions	Eurofins CRL, USA	3	Acid digestion, ICP-OES	Pre-validated	Validated in study
28. Sugars	Eurofins CRL, USA	3	Extraction, colorimetric/CFA	Pre-validated	Pre-validated
29. Volatile organic compounds	Labstat Int.	3	Extraction, GC-MS	Pre-validated	Pre-validated
30. Water	Eurofins CRL, USA	3	Karl Fischer analysis	Pre-validated	Pre-validated

**Table 3:** Summary of measurands present at quantifiable levels (per-pouch) in ONPs and snus

Measurand (unit)	ONPs			Snus		Measurand (unit)	ONPs			Snus	
	No.^1^	Min	Max	Min	Max		No.^1^	Min	Max	Min	Max
*Major measurands*						*Oxygenated analytes*					
Mean mass (mg)	11	260	1285	889	999.7	Acetaldehyde (µg)	6	NQ	0.188	6.22	8.08
Water (%)	11	4.11	49.4	48.6	49.7	Acrolein (µg)	2	BDL	0.283	BDL	NQ
pH	11	8.51	9.07	7.44	8.07	Formaldehyde (µg)	11	0.333	2.69	1.01	1.17
*Hydrocarbons & PAHs*						Glycerol (mg)	1	BDL	68.4	BDL	BDL
Toluene (ng)	3	BDL	2.987	1.85	3.8	Propylene glycol (mg)	6	BDL	12.2	24.6	24.6
1-Methylnaphthalene (ng)	11	1.53	27.1	5.47	6.5	*Microbiology*					
2-Methylnaphthalene (ng)	11	2.343	38.4	3.42	4.42	Total bacterial count (CFU)	0	< RL	< RL	< RL	41.5
Acenaphthene (ng)	11	0.181	2.21	0.451	0.661	Mould count (CFU)	0	< RL	< RL	< RL	91.6
Acenaphthylene (ng)	11	0.225	2.03	0.604	0.803	*Nitrogenous analytes*					
Anthracene (ng)	3	BDL	0.948	0.823	1.02	Total alkaloids (mg)	11	4.793	29.81	6.12	7.41
Benzo[a]anthracene (ng)	2	BDL	0.107	1.27	1.28	Anabasine (µg)	3	BDL	11.67	22.7	29.7
Benzo[b]fluoranthene (ng)	6	BDL	0.155	1.18	1.26	Anatabine (µg)	3	BDL	11.43	64.4	91.1
Benzo[g, h,i]perylene (ng)	11	0.108	2.33	0.752	0.954	β-Nicotyrine (µg)	4	NQ	1.503	13.4	21.7
Benzo[j]fluoranthene (ng)	0	BDL	BDL	0.783	0.865	Cotinine (µg)	10	NQ	1.393	36	45.1
Benzo[k]fluoranthene (ng)	1	BDL	0.224	0.534	0.54	Myosmine (µg)	10	NQ	9.5	5.6	9.09
Chrysene (ng)	4	NQ	0.132	2.09	2.5	Nicotine-1’-oxide (µg)	11	4.4	134	181	182.7
Dibenz[a, h]anthracene (ng)	4	BDL	0.087	0.199	0.202	Nornicotine (µg)	11	1.3	27.1	81	121
Fluoranthene (ng)	7	BDL	1.135	8.61	9.95	Nicotine (mg)	11	5.367	20.3	5.87	7.15
Fluorene (ng)	11	0.095	4.66	0.553	0.665	Acrylamide (ng)	5	NQ	27.3	142	191
Indeno[1,2,3-cd]pyrene (ng)	7	BDL	0.285	0.599	0.803	Ethyl carbamate (ng)	3	BDL	66.8	BDL	13.5
Naphthalene (ng)	11	3.36	31.8	10.9	12.9	Nitrate (mg)	0	NQ	NQ	5.62	6.97
Phenanthrene (ng)	11	0.382	8.73	8.28	11.2	*Nitrosamines & Nitrosacids*					
Pyrene (ng)	3	BDL	1.29	6.03	6.847	NDBzA (ng)	1	BDL	3.34	BDL	BDL
*Inorganics*						NDELA (ng)	5	NQ	3.79	4.5	5.5
Ash (g)	11	0.0168	0.0722	0.122	0.135	NAB (ng)	0	BDL	BDL	11.4	11.8
Arsenic (ng)	7	NQ	7.45	41.4	58.6	NAT (ng)	0	BDL	BDL	169	192
Beryllium (ng)	2	NQ	0.89	6.47	7.05	NNK (ng)	0	BDL	BDL	74.6	79.1
Cadmium (ng)	0	NQ	NQ	216	219	NNN (ng)	0	BDL	BDL	226	262
Chromium (ng)	0	NQ	NQ	298	354	MNPA (ug)	0	< RL	< RL	0.101	0.119
Lead (ng)	1	NQ	26.87	99.1	127	NPRO (µg)	0	< RL	< RL	0.12	0.251
Mercury (ng)	0	NQ	NQ	2.56	3.34						
Nickel (ng)	5	NQ	85.7	620	756						
Selenium (ng)	0	NQ	NQ	NQ	98.7						
Sodium (mg)	11	6.41	26.7	23	23.4						
Carbon-14 (Bq)	11	0.006	0.025	BDL	BDL						
Polonium-210 (Bq)	1	BDL	0.00139	0.0032	0.004						
Potassium-40 (Bq)	0	BDL	BDL	0.515	0.602						

BDL, below detection limit; NQ, not quantified, < RL, below reporting limit

^1^ Number quantified among 11 ONPs, except for NPRO, which was quantified in 8 ONPs

**Table 4:** Percentage difference in contents of WHO TobReg priority measurands among ONPs, snus and cigarette smoke

Measurand	Difference between ONPs and snus (%)	Difference between ONPs and cigarette smoke (%)	Difference between snus and cigarette smoke (%)
*Toxicants recommended for mandated lowering* ^1^			
1,3-Butadiene	ND in either	–99.9	–99.9
Acetaldehyde	–98.7	–99.9	–99.6
Acrolein	+ 17.1	–99.9	–99.9
Benzene	ND in either	–99.9	–99.9
Benzo[a]pyrene	NQ in either	–99.9	–99.9
CO	NM in either	NM in ONPs	NM in snus
Formaldehyde	–16.6	–98.7	–98.4
NNK	–99.3	–99.7	–63.0
NNN	–99.9	–99.8	+ 28.0
*Toxicants considered high priority for disclosure and monitoring* ^1^			
2-Aminonaphthalene	ND in either	–97.7	–97.1
4-aminobiphenyl	NQ in either in either	–98.2	–90.2
Acrylonitrile	ND in either	–99.9	–99.9
Cadmium	–99.6	–98.9	+ 145.5
Catechol	NM in either	NM in ONPs	NM in snus
Crotonaldehyde	ND in either	–99.9	–99.9
HCN	NM in either	NM in ONPs	NM in snus
Hydroquinone	NM in either	NM in ONPs	NM in snus
NOx	NM in either	NM in ONPs	NM in snus

ND, not detected; NQ, not quantified; NM, not measured

^1^ Ref. [56]

**Table 5:** Compilation of previously reported ONP chemical contents

Study	Wagner et al. 2020 [29]	Azzopardi et al. 2021 [20]	Avagayan et al. 2021 [30]	Jablonski et al. 2022 [31]		Jablonski et al. 2022 [31]	Miller et al. 2022 [32]	Back et al. 2023 [33]	Mallock et al. 2022, 2024 [34]
	on! Mint, 5 nic strengths	Lyft, 4 flavours	4 ONPs	13 ONPs		13 ONPs	9 on! ONPs	2 Zyn ONPs	46 ONPs
Units	Per portion	Per gram	Per gram	Per portion		Per gram	Per portion	Per gram	Per pouch
Notes				Non-plant ONPs only (x/13) = no of obs					
Mass (g)		0.7		0.26–0.68		0.26–0.68		0.4–0.8	0.305–1.246
Moisture (%)		48						3–37	
pH	8.2–8.81	8.6						8.3	5.5–10.5
Acetaldehyde (µg)	NQ (< 0.05)–0.063	NQ (< 1)		ND (9/13)–0.137		ND (< 0.063)–0.557	0.430 (0.262–0.774)	NQ (< 1)	
Formaldehyde (µg)	0.1–0.111	NQ (< 1)–1.13		0.124–5.24		0.326–13.4	NQ (< 0.1)–0.249	1.5–10.3	
Acrolein (µg)		NQ (< 0.05)							
Crotonaldehyde (µg)	NQ (< 0.025)	NQ (< 0.05)		ND		ND (< 0.022)	NQ (< 0.1)	NQ (< 0.05)	
As (ng)	NQ (< 5)	NQ (< 50)		ND (9/13)–NQ		ND (< 3)–NQ (< 10)	NQ – (< 20)	NQ (< 50)	
Be (ng)		–		ND (9/13)–NQ		ND (< 0.25)–NQ (< 2.5)		NQ (< 50)	
Cd (ng)	NQ (< 5)	< 10		ND (10/13)–NQ		ND (< 0.5)–NQ (< 5)	NQ (< 20)	NQ (< 50)	
Cr (ng)		NQ (< 50)–80		11.2–177		22.5–274		99–160	
Co (ng)		–		NQ(5/13)–5.47		NQ (< 6)–10.4			
Pb (ng)		NQ (< 20)		1.59–56		3.88–83.2		NQ (< 20)	
Mercury (ng)		NQ (< 20)						NQ (< 20)	
Ni (ng)		NQ (< 1)		ND (1/13)–89.1		ND (< 20)–138		NQ (< 50)–67	
Se (ng)		–		ND (6/13)–NQ		ND (< 4.6) – NQ (< 20)		NQ (< 50)	
210-Po (mBq/g)								NQ (< 5)	
235-U								NQ (< 0.02)	
238-U								NQ (< 0.25)	
Nicotine (mg)	8.32 (highest sample)	0.58%–1.7%		4–12		3.1–8.0	8.97 (5.65–12.71)	7.63–12	ND–47.5
Free/unprotonated Nicotine (mg)	5.23 (highest sample)	–					6.83 (3.81–10.02)	4.87–7.87	(0) 0.3–100%
Nicotine -N-oxide (µg)			2.6–74						
Nornicotine (µg)			ND(< 0.46)–9.8					NQ (< 50)	
Anabasine (µg)			ND(0.08)–2.9					NQ (< 20)	
Anatabine (µg)			ND(< 0.18) – 1.4						
Myosmine (µg)			ND(< 0.36)–4.3						
β-Nicotyrine (µg)			ND(< 0.61)						
Cotinine (µg)			ND(< 0.1)–1.2						
Nitrite (µg)		NQ (< 1)		ND (1/13) – 0.509		ND (< 0.033)–1.55		NQ (< 1)	
NAB (ng)		NQ (< 10)		ND		ND (< 0.51)		NQ (< 10)	ND (< 0.34; 21/26)–5.6
NAT (ng)		NQ (< 10)		ND		ND (< 1.3)		NQ (< 10)	ND (< 0.22; 20/26)–2.7
NNK (ng)	ND (< 40)	NQ (< 10)		ND		ND (< 1.3)	NQ (< 4)	NQ (< 10)	ND(< 0.12;23/26)–5.4
NNN (ng)	ND (< 40)	NQ (< 10)		ND–NQ (2/13)		ND (< 1.3)–NQ (< 13)	NQ (< 4)	NQ (< 10)	ND(< 0.12;2/26)–13
NDMA (ng)		NQ (< 0.2)						NQ (< 0.2)	
NDELA (ng)								NQ (< 25)	
NPIP (ng)								NQ (< 10)	
NPYR (mg)								NQ (< 10)	
NSAR (ng)								NQ (< 25)	
1,3-Butadiene (ng)		NQ (< 20)							
Benzene (ng)		NQ (< 5)							
B[a]P (ng)	ND (< 0.13)	NQ (< 1)		ND		ND(< 0.06)	NQ (< 0.5)	NQ (< 1)	
Benz[a]anthracene (ng)								NQ (< 30)	
Benzo[b, k]fluoranthene (ng)								NQ (< 30)	
Chrysene (ng)								NQ (< 30)	
Dibenz[a, h]anthracene (ng)								NQ (< 30)	
Indeno[1,2,3-cd]pyrene (ng)								NQ (< 30)	
Naphthalene (ng)								NQ (< 30)	
Aflatoxin B1 (ng)		NQ (< 1)						NQ (< 1)	
Aflatoxin B2 (ng)		NQ (< 1)						NQ (< 1)	
Aflatoxin G1 (ng)		NQ (< 1)						NQ (< 1)	
Aflatoxin G2 (ng)		NQ (< 1)						NQ (< 1)	
Ochratoxin A		NQ (< 0.5)						NQ (< 0.5)	
Ammonia (µg)				Non-plant ONPs only		Non-plant ONPs only		62–66	
Coumarin (µg)								NQ (< 0.05)	
Ethyl carbamate (ng)								NQ (< 30)	

ND, not detected; NQ. Not quantified

**Fig. 1:**
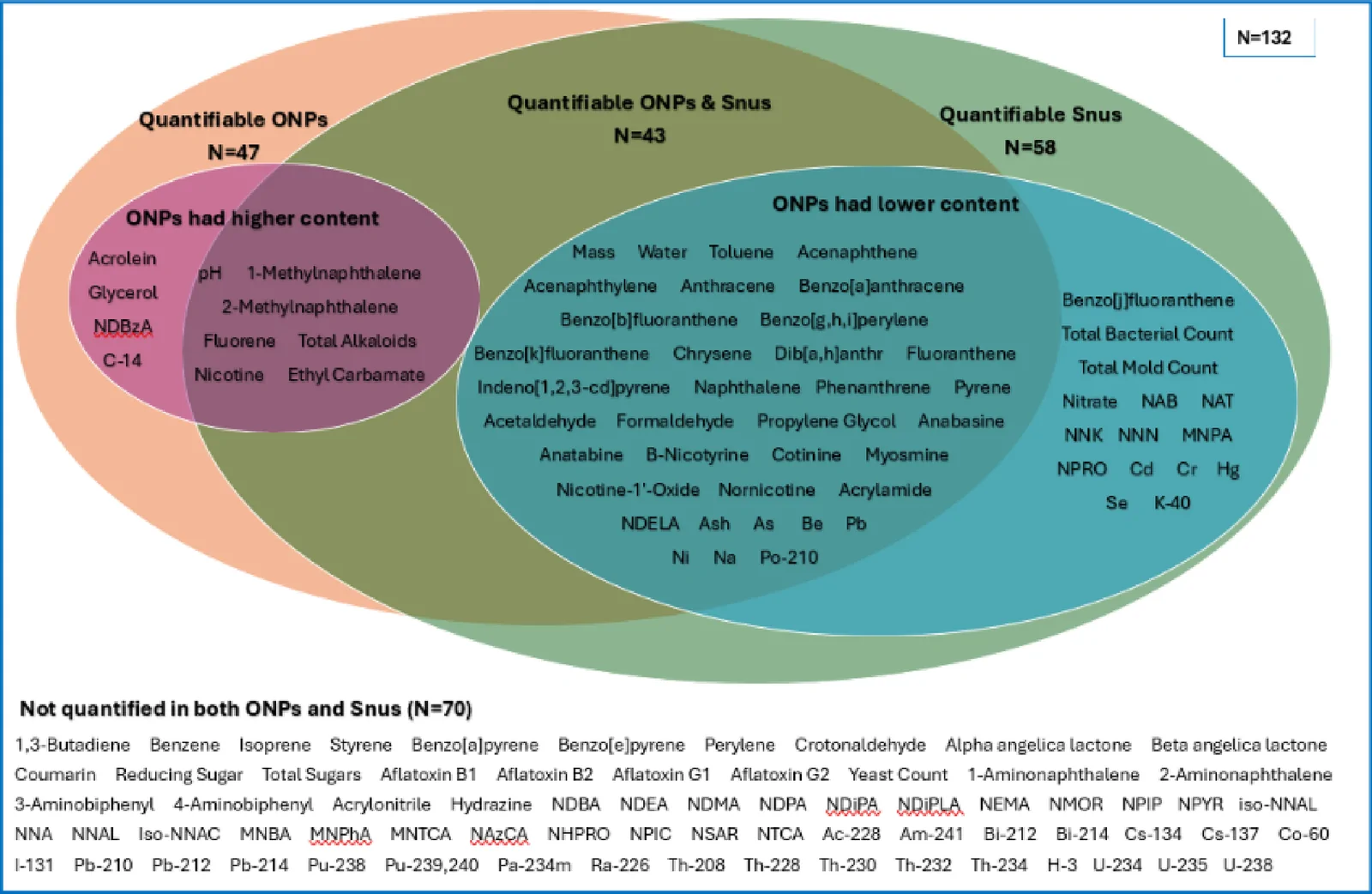
Venn diagram summarizing the numbers of compounds quantified in ONPs and snus

**Fig. 2:**
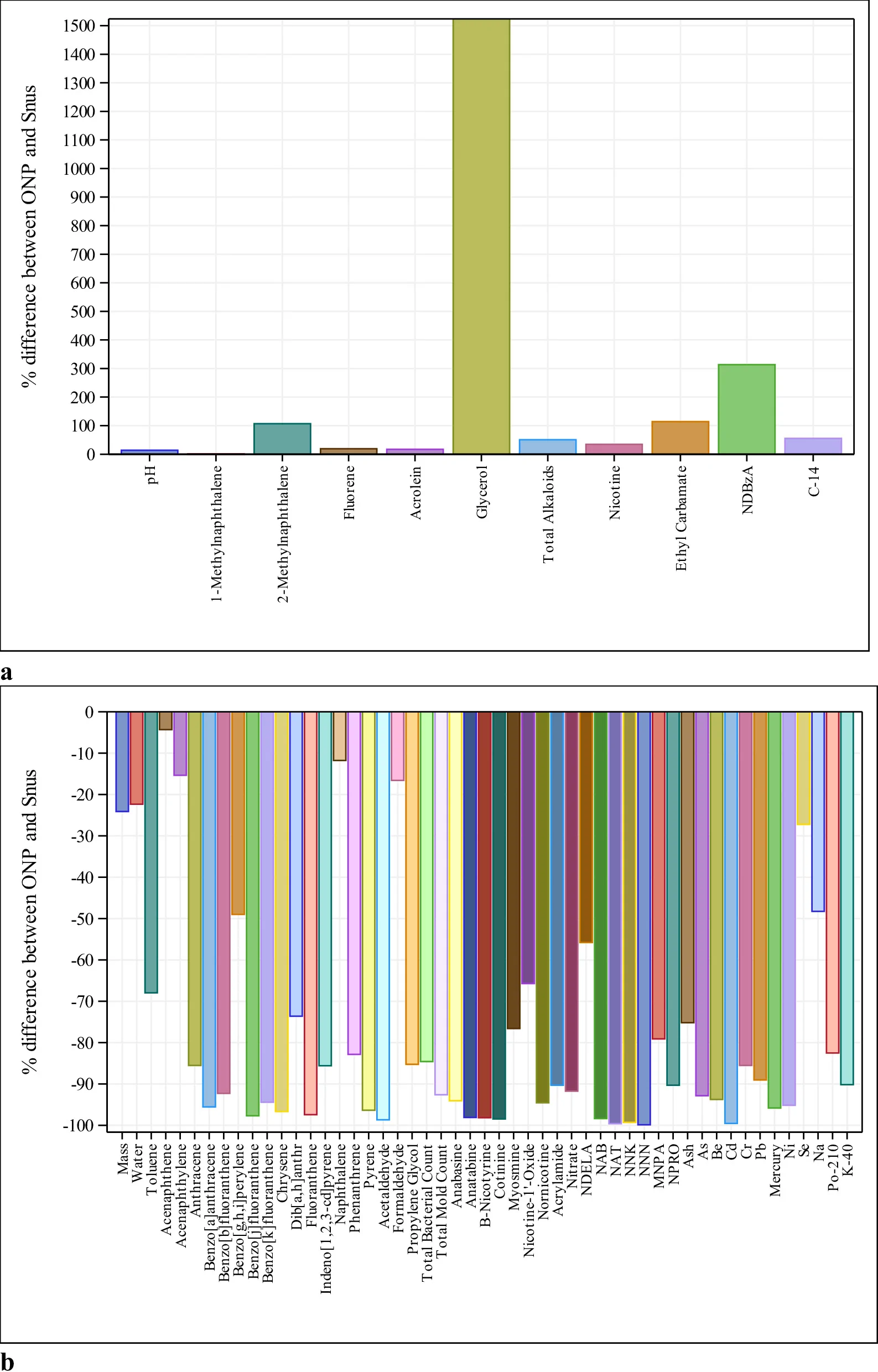
Comparison of ONP and snus contents.** A** Measurands at higher levels (averaged across study samples) in the study ONPs than snus samples** B** Measurands at lower levels (averaged across study samples) in the study ONPs than snus samples

**Fig. 3:**
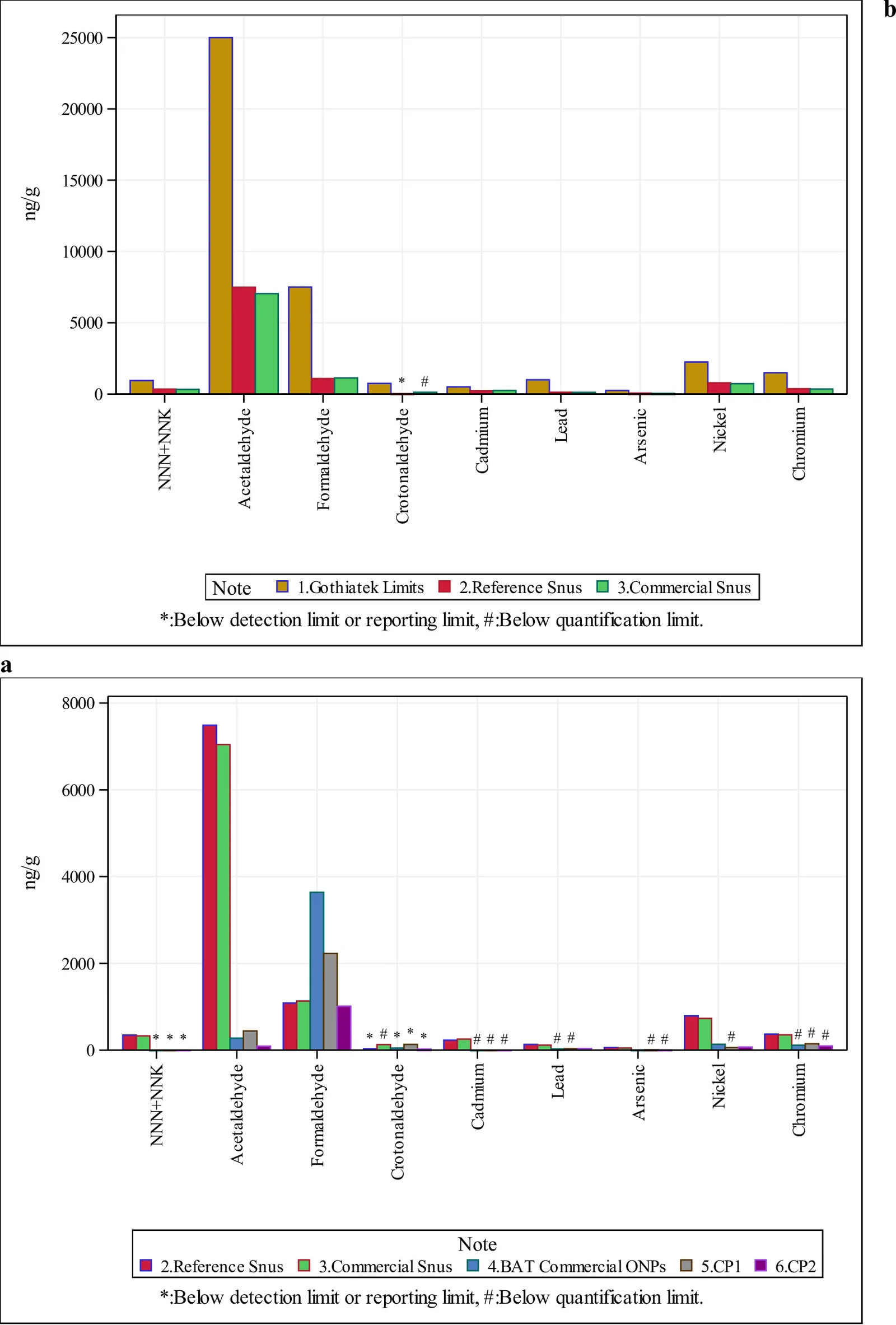
Gothiatek^®^ limits for analytes having limits > 20 ng/g. **A** Gothiatek^®^ limits vs. Study Snus at limits > 20 ng/g. **B** Study Snus, BAT ONPs, CP1 and CP2 analytes that had Gothiatek^®^ limits > 20 ng/g

**Fig. 4:**
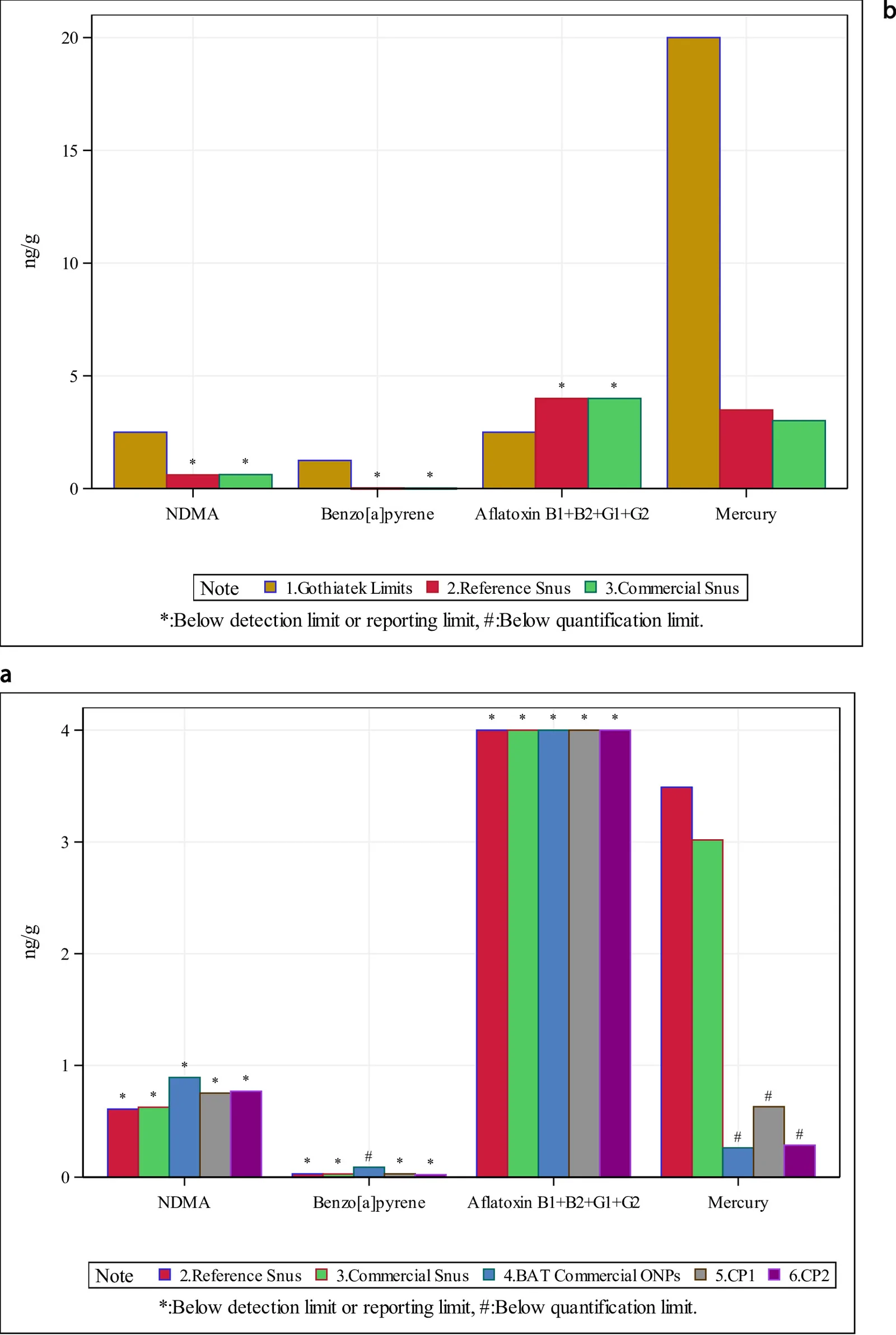
Gothiatek^®^ limits for analytes having limits ≤ = 20 ng/g. **A** Gothiatek^®^ limits vs. Study Snus at limits < = 20 ng/g. **B** Study Snus, BAT ONPs, CP1 and CP2 analytes that had Gothiatek^®^ limits < = 20 ng/g

**Fig. 5:**
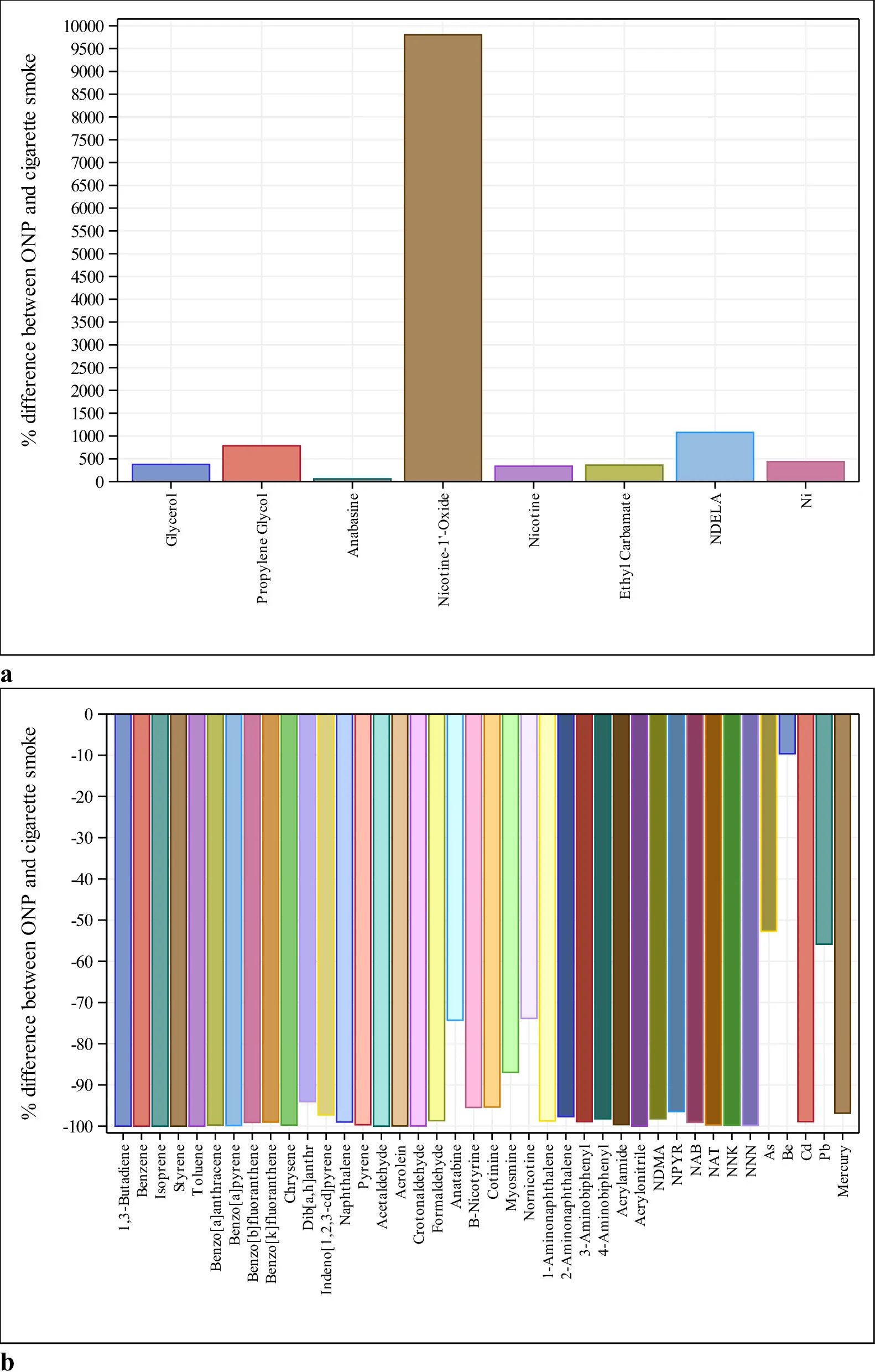
Comparison of per-cigarette smoke emissions with mean per-pouch ONP contents.** A** Measurands at higher levels (averaged across study samples) in the study ONPs than 1R6F reference cigarette smoke.** B** Measurands at lower levels (averaged across study samples) in the study ONPs than 1R6F reference cigarette smoke

**Fig. 6:**
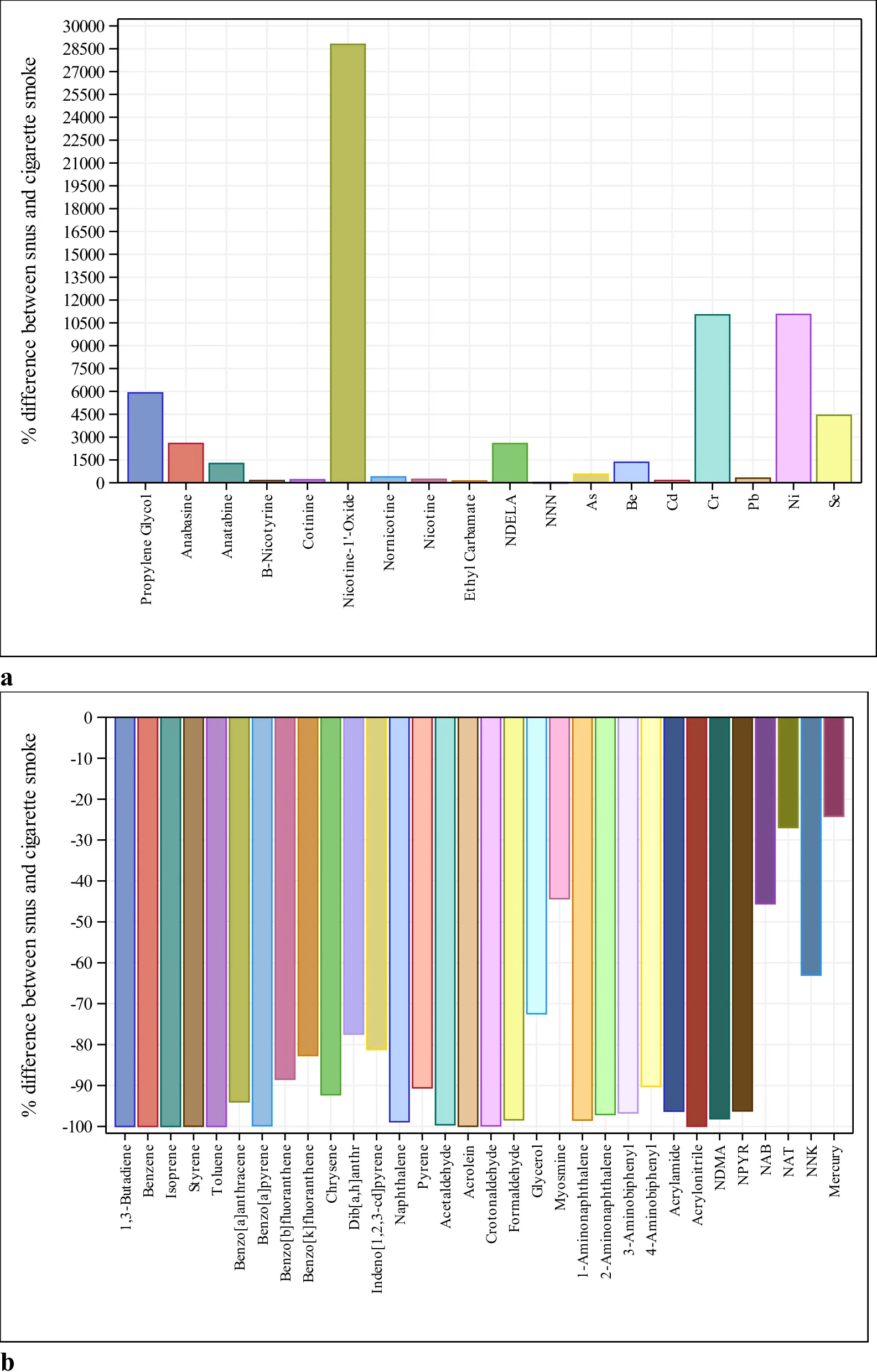
Comparison of per-cigarette smoke emissions with mean per-pouch snus contents.** A** Measurands at higher levels (averaged across study samples) in both snus products tested in this study than 1R6F reference cigarette smoke.** B** Measurands at lower levels (averaged across study samples) in both snus products tested in this study than 1R6F reference cigarette smoke

### Comparing the toxicities of snus and ONP contents

As described above, several compounds were present in different quantities in Snus and ONPs. A number of these compounds have well-defined toxicological properties in humans. Considering cancer risk, IARC categorises the carcinogenicity of compounds into “carcinogenic to humans” (Group1), “probably carcinogenic to humans” (Group 2A), ‘possibly carcinogenic to humans” (Group 2B), “not classifiable as to carcinogenicity to humans” (Group 3), and “probably not carcinogenic to humans” (Group 4). We examined the IARC classifications of the compounds that differed in content between Snus and ONPs.

Our measurements showed that snus contained higher levels of several IARC Group 1 carcinogens than the ONPs. This included arsenic, beryllium, cadmium, formaldehyde, ^40^K, NNN, NNK, and ^210^Po; levels of Cr and Ni were also higher in Snus, and some chemical forms of these metals are Group 1 carcinogens. In contrast, ONPs contained only one Group 1 carcinogen at higher levels than snus, ^14^C.

Considering IARC Group 2 A, probable carcinogens, snus contained higher levels of dibenz[a, h]anthracene, acrylamide and nitrate, whilst ONPs contained higher levels of acrolein and ethyl carbamate. In terms of possible carcinogens (IARC Group 2B), snus contained higher levels of acetaldehyde, anthracene, benzo[b]-, benzo[j]-, and benzo[k]-fluoranthene, chrysene, indeno[1,2,3-cd]pyrene, lead, mercury, naphthalene and NDELA.

Overall, therefore, it appears that snus contains higher levels of a wider range of established and possible carcinogens to humans than ONPs. Whilst it is challenging to extrapolate the toxicology of individual compounds to that of a complex mixture, these observations suggest that the cancer risk of ONPs may be comparable or lower than that of snus.

One compound that was notably higher on average in these ONPs than in the study snus samples is nicotine. Nicotine is not considered carcinogenic [48], but is addictive, and can cause several potentially adverse physiological effects amongst those exposed to it. An FDA Technical Project Lead (TPL) Review of Nicotine Pouch PMTAs [73] noted that long-term nicotine exposure has been associated with adverse cardiovascular effects, systemic inflammation, gastrointestinal effects, and effects on bone growth.

There is limited data currently available in the literature to identify whether these nicotine-related adverse physiological effects are observed amongst ONP-users. In terms of health effects associated with ONP use, Farsalinos [74] reviewed currently available literature and concluded that ONP use can cause acute and longer-term mucosal irritation compared to not using any nicotine/tobacco product. The US FDA [52] noted that such effects can include parakeratosis, edema, and chronic inflammation. However, Farsalinos [74] also concluded that the prevalence and severity of oral health problems resulting from ONP use were lower than those occurring amongst smokers or snus users. Hartmann-Boyce et al. [2025] reviewed three published short-term Randomised Control Trials (RCT) on ONPs and found no incidence of serious adverse events, whilst US FDA [73] noted that reported adverse events in one RCT were mild to moderate in severity and with no product-specific serious or unexpected AEs reported.

It is challenging to draw robust conclusions concerning comparative risk profiles of different product types from chemical content data alone [7]. However, the lower numbers of quantifiable measurands in ONPs than snus, the observation that few measurands were higher in ONPs while many were substantially lower, the presence of fewer IARC Group 1 and 2B compounds in ONPs than snus, and compliance with the GOTHIATEK^®^ standard, together suggests that ONPs might potentially offer a reduced risk profile to exclusive users of ONPs as compared with use of snus.

### Study limitations

Other factors must be recognised when considering the implications for relative toxicant exposure from ONPs, snus and cigarette smoke. First, usage patterns will vary among both smokers and ONP users; thus, individual exposure may differ significantly from the chemical data obtained in this study under standardised laboratory conditions. Second, relying solely on targeted analyses to characterise a product’s chemical composition has limitations for the understanding generated, as the data are inherently limited to the chemicals selected by the study design. An additional complementary approach is the use of non-targeted chemical scans; these can provide substantially greater volumes of data on chemical composition than those of targeted analyses, albeit with challenges in compound identification, quantification and method sensitivity. Third, product content data of the kind provided in this study indicates the maximum quantity of measurands entering the consumer’s body or that the consumer could be exposed to. However, smokers exhale cigarette smoke and oral product users remove their pouch after use. Both usage patterns will reduce the uptake of toxicants compared to the product content or amount initially entering the body. Fourth, it is challenging to accurately compare the impact of chemical data from products as different as oral products and inhalation products. The primary exposure sites within the body differ between cigarette smoke (respiratory tract and lungs) and ONPs/snus (oral mucosa/GI tract) and will influence the potential disease outcomes arising from such exposures. Finally, to compare absolute exposure or risks between products, in addition to the factors above, knowledge of product consumption patterns, such as the numbers of products used per day, is required to more accurately estimate exposure.

### ONPs and tobacco harm reduction

Tobacco Harm Reduction was defined by the Institute of Medicine (IOM) as “minimising harms and decreasing total mortality and morbidity, without completely eliminating tobacco and nicotine use” [75]. Tobacco harm reduction involves providing those unwilling or unable to quit tobacco products with less harmful nicotine-containing products for continued use [76].

The challenge in demonstrating THR associated with use of an alternative nicotine product is that the harms associated with conventional tobacco use develop slowly, and a time scale of decades of product use might be needed to fully establish the extent and effectiveness of THR.

As a first step in indicating the potential for THR, the IOM developed the concept of a “Potentially Reduced Exposure Product” (PREP), a product designed to lower intake of toxicants compared to traditional tobacco products. The IOM also provided a scientific framework to comprehensively evaluate whether candidate-PREPs might be associated with reduced disease risks compared with conventional tobacco products. The report identified the importance of assessing chemical content/emissions, toxicity, appeal, addictiveness, and consumer perception of a candidate PREP to determine its likely public health impact [75].

The FDA has since introduced the concept of a “Modified Risk Tobacco Product (MRTP)” which is a product “sold or distributed for use to reduce harm or the risk of tobacco-related disease associated with commercially marketed tobacco products” [77].

The 2009 US Family Smoking Prevention and Tobacco Control Act provided a regulatory framework under which tobacco companies could introduce tobacco products with lower-exposure or risk claims after scientific review and upon obtaining an FDA marketing order.

The FDA has identified the scientific evidence required to demonstrate that the product may reduce harm or exposure compared to conventional cigarettes; the evidence base examines both the product and its impact on public health [78]. Required product information includes comprehensive quantitative HPHC data, clinical biomarker data reflecting human exposure to toxins, and in vitro and in vivo studies demonstrating the product’s reduced toxicity compared to combusted cigarettes.

The FDA also requires consumer perception data, gateway effect studies and behavioural data on how consumers use the product in real-world settings. Estimates of the potential population health benefit require quantitative epidemiological model estimates and data on switching rates, examining the extent to which adult smokers completely switch away from combusted cigarettes, rather than dual-use. Finally, environmental impact assessment studies and post-market studies to continuously monitor consumer understanding, product use, and long-term health outcomes.

In the context of the IOM and FDA evidence frameworks, the data provided in this study only addresses the first stages of the required product data. Further extensive studies, of the form identified by IOM and FDA, would be required to more fully characterise the potential of ONPs to effect THR at the population level. However, the data from this study provide a good starting point for future studies of this kind.

## Conclusion

Our wide-ranging study (132 measurands) of the chemical contents of 11 ONPs, one commercially available Swedish snus and a reference snus has shown slightly fewer quantifiable measurands in ONPs than snus. On a quantitative basis, while a small number of analytes were present at higher concentrations in some ONPs, the levels of most measurands were substantially lower in ONPs than snus.

Comparing the chemical contents of ONPs with cigarette smoke showed almost all comparators were substantially lower in ONPs. A similar comparison of snus contents with cigarette smoke showed similar results to that of ONPs, albeit the differences between snus and cigarette smoke were less pronounced.

The smaller numbers of measurands in ONPs compared to snus, along with our observations that many of these compounds were present at significantly lower levels in ONPs, suggest that ONPs may offer a comparable or potentially reduced health risk alternative to exclusive users of ONPs in comparison with continued use of snus. Similarly, comparing the contents of ONP and snus products with cigarette smoke supports the viewpoint that Swedish snus presents lower health risks to the user than smoking cigarettes, and suggests that exclusively using ONPs may also provide a potentially reduced risk alternative as compared to continued cigarette smoking.

In relation to the important first step of chemically characterising ONPs in contrast with cigarettes and snus, the ONPs studied have overall shown similar or lower levels of the measured toxicants compared with the snus products tested, suggesting an initial positioning of ONPs similar to snus on the tobacco and nicotine risk continuum. Further investigations are required, such as behavioural, chemical, toxicological and clinical studies and comparison with products with existing long-term epidemiology to more accurately establish ONPs position on the tobacco and nicotine risk continuum.

## Supplementary Information

Below is the link to the electronic supplementary material.

Supplementary Material 1. Analytical methods.

Supplementary Material 2. Table S1: LODs and LOQs. Table S2: Measured contents. Table S3: Comparison to 1S4. Table S4: Comparison to General Classic Blend White Portion Large Snus. Table S5: Comparison to GOTHIATEK^®^ limits. Table S6: Comparison to 1R6F.

## Data Availability

The authors declare that the data supporting the findings of this study are available within the paper and its Supplementary Information files. Should any raw data files be needed in another format they are available from the corresponding author upon reasonable request.
