# Supplementary material for: Toxicant contents of oral nicotine pouches: comparative quantitative analysis with Swedish snus and cigarette smoke
Source: BMC Chem. 2026 Aug 14;20(1):219. doi: 10.1186/s13065-026-01895-x (PMC13753406; doi:10.1186/s13065-026-01895-x)
Supplement: Supplementary file 1 [file 13065_2026_1895_MOESM1_ESM.docx]

**Supplementary File 1: Analytical Methods used in this study, and validation data.**

**Section S1. Analytical Methods:**

***1.1 Acrylamide***

Acrylamide was measured by Eurofins CRL (Winston-Salem, NC, USA). In brief, ground and homogenized samples were extracted by machine-shaking with a magnesium sulfate/sodium chloride salt extraction packet containing internal standard. After centrifugation, the supernatant was subjected to evaporation using a Turbovap solvent evaporation system. The residue was redissolved in water and analysed by liquid chromatography–tandem mass spectrometry (LC–MS/MS) using a BEH Shield RP18 column (1.7 mm particle size, 2.1 × 100 mm) or equivalent.

***1.2 Aflatoxins***

Aflatoxins B1, B2, G1 and G2 were measured by high-performance liquid chromatography (HPLC) at Eurofins Food and Feed Testing Sweden AB (Lidköping, Sweden). Finely comminuted samples were homogenised and extracted with methanol/water. The extract was then filtered and diluted with phosphate-buffered saline (PBS). An ASPEC®

automatic liquid handler was used for extract dilution, mixing, HPLC injection, and elution of the aflatoxins. Analysis was performed by using bromine post-column derivatisation (cobra cell) and fluorescence detection.

***1.3 Alkaloids***

Eurofins CRL measured individual alkaloids contents. Samples were machine-shaken in 10 mL of ultrapure water for 90 min. Sodium hydroxide was added, the solution was allowed to stand, and then re-shaken. After separating the layers, the top layer was transferred to a gas chromatography (GC) vial for analysis by GC–MS using a DB-35MS column.

Eurofins CRL measured total alkaloid contents. Samples were extracted in an acetic acid, methanol and water solution. The total alkaloid content was determined by reacting the extract with sulfanilic acid and cyanogen chloride at 40 °C. Cyanogen chloride was generated in situ by reacting potassium cyanide and chloramine-T. The resulting colour was measured at 480 nm on a continuous flow analyser.

***1.4 Aromatic amines***

1-Aminonaphthalene, 2-aminonaphthalene, 4-aminobiphenyl and 3-aminobiphenyl were measured by Labstat International (Kitchener, Canada). Ground and homogenized samples were combined with an internal standard (4-aminobiphenyl-d9) and extracted by shaking with dilute hydrochloric acid. After filtration, the extract was subjected to a liquid–liquid extraction process, followed by derivatization using pentafluoropropionic acid anhydride and trimethylamine. The derivatized extract was subjected to magnesium silicate solid-phase extraction (SPE) and concentrated before analysis by electron impact GC–MS using a non-polar fused-silica capillary column.

***1.5 Angelica lactones***

The angelica lactones were measured by UK-based external laboratory. Samples were weighed directly into a headspace vial. Magnesium sulphate was added followed by acetophenone-d8 internal standard and a calibration standard solution. The resulting samples were analysed by headspace GC–MS using an Agilent DB-624 UI column.

***1.6 Ash***

Inorganic content was measured by Eurofins Food & Feed Testing Sweden AB using gravimetric estimation. Samples were weighed and then heated in air at 550 ± 25 °C for 16–18 hours. They were cooled in a desiccator before weighing the remaining ash content.

***1.7 Carbonyls***

Labstat International measured acetaldehyde, acrolein, crotonaldehyde and formaldehyde contents of the samples. Analytes were extracted with ultrapure water containing internal standards (deuterated equivalents of the target analytes), derivatised using o-(2,3,4,5,6-pentafluorobenzyl)-hydroxylamine hydrochloride (PFBHA), acidified and extracted with toluene. They were quantified using GC-MS with selected ion monitoring (SIM).

***1.8 Coumarin***

Coumarin contents were measured by Labstat International. Samples were extracted with an ethanol/ultrapure water solution containing internal standard (coumarin-d4) using a wrist action shaker; the crude extract was filtered and further diluted using methanol/ultrapure water. The resulting filtrate was analysed by LC–MS/MS using a reversed-phase C18 guard column (or equivalent) and C8 column for separation and positive electrospray ionisation (ESI) and multiple reaction monitoring (MRM) for detection.

***1.9 Ethyl carbamate***

Labstat International measured ethyl carbamate contents of the samples. Extraction took place by mixing the sample with deuterated ethyl carbamate internal standard solution followed by shaking with ultrapure water. After centrifugation, the supernatant was filtered and subjected to solid-phase supported liquid–liquid extraction using cartridges containing diatomaceous earth, dichloromethane and ethyl acetate. The concentrated eluate was analysed by GC–MS (operated in SIM mode) using a polyethylene glycol (PEG) capillary column for separation.

***1.10 Humectants***

Propylene glycol and glycerol were analysed by Labstat International using a standard method [1]. Samples were machine-shaken with methanol solution containing internal standard (1,3-butanediol). After centrifugation, the supernatant was analysed by GC with flame ionization detection (GC-FID) using a PEG capillary column or equivalent.

***1.11 Hydrazine***

Hydrazine was measured by UK-based external laboratory. Samples were placed in headspace vials, to which sodium chloride, water and acetone were added. Derivatised samples were analysed for acetone azine by GC–MS with a PAL RTC Autosampler and using a 60m Restek 624-Si MS column.

***1.12 Mass***

Product masses were measured gravimetrically using a calibrated four-decimal-place balance. Product masses were measured by all measurement laboratories, and a total of 36 replicates were obtained. The quoted mass values are the mean value of these 36 replicates.

***1.13 Metals and metalloids***

Elemental analysis was conducted by Eurofins CRL. Samples were digested in nitric acid using closed-vessel microwave digestion and then diluted in ultrapure water after the addition of internal standard. Arsenic, beryllium, cadmium, chromium, cobalt, lead, mercury, nickel, and selenium were analysed by inductively coupled plasma MS equipped with reaction cell technology to remove isobaric interference caused by isotopes of different elements forming atomic ions with the same mass/charge ratio as the analyte of interest.

***1.14 Mercury***

Mercury was quantified by direct mercury analysis conducted by Eurofins CRL as follows. Samples were placed in a decomposition furnace to release mercury vapor, which was reduced to elemental mercury and selectively trapped by a gold amalgamator. Next, the amalgamator was heated to release the mercury into a fixed wavelength double beam atomic absorption spectrophotometer. Mercury content was quantified by absorbance at 253.65 nm.

***1.15 Microbiological counts***

Total bacterial count, total yeast and mould count were measured by Eurofins CRL. Microorganisms were differentiated through the types of medium and incubation conditions (i.e., time, temperature) used for processing. Samples were plated under conditions favourable for the target microorganism, together with a positive control organism and an uninoculated plate as a negative control. Colonies on the plates were enumerated for data analysis.

***1.16 Nicotine***

Eurofins CRL measured the nicotine contents of the samples. A minimum of 1 g of whole pouch material was slit open and conditioned using sodium hydroxide solution prior to extraction using a solution of quinoline (internal standard) in methyl tert-butyl ether. After being allowed to separate for 1 hour, the top fraction of the extract was analysed by GC-FID with separation via a DB-35 column.

**1.17. Nicotine-related compounds**

Nicotine-N’-oxide, cotinine, nornicotine, anatabine, myosmine, anabasine and β-nicotyrine contents were measured by Labstat International. Samples were machine-shaken with ammonium acetate solution containing internal standard (cotinine-d3, nornicotine-d4, β-nicotyrine-d3, anatabine-d4, anabasine-d4, nicotine-*N*-oxide-d3 and myosmine-d4). After centrifugation, the supernatant was analysed by LC–MS/MS in positive ESI and MRM using a reversed-phase C18 column or equivalent for separation.

***1.18 Nitrate nitrogen***

Nitrate nitrogen was measured by Eurofins CRL. Aqueous extracts of samples were subjected to a cadmium reduction column to reduce the nitrate to nitrite in situ. The nitrite (original content plus reduced nitrite) was determined by reaction with sulfanilamide and naphthylethylenediamine dihydrochloride to form a coloured adduct. Absorbance was measured at 540 nm and the content determined from calibration using a linear through zero regression.

***1.19 Nitrosamines (TSNAs)***

Four TSNAs, *N*-nitrosoanabasine (NAB), *N*-nitrosoanatabine (NAT), 4-(methylnitrosamino)-1-(3-pyridyl)-1-butanone (NNK) and *N*-nitrosonornicotine (NNN), were measured by Labstat International. Samples were extracted by shaking with ammonium acetate solution containing internal standards (NNN-d4, NAT-d4, NAB-d4 or NNK-d4). Aliquots were syringe-filtered and analysed by LC–MS/MS set up in positive ESI and MRM using a C18 guard column and a C18 column or equivalent.

**1.20 Nitrosamines (NNA, NNAL, iso-NNAL)**

Analysis of 4-(methylnitrosamino)-1-(3-pyridyl)-1-butanol (NNAL), 4-(methylnitrosamino)-4-(3-pyridyl)-1-butanol (iso-NNAL) and 1-(*N*-methyl-*N*-nitrosamino)-1-(3-pyridinyl)-4-butanal (NNA) was conducted by UK-based external laboratory, samples were fortified by adding internal standards (deuterated analogues of target analytes) to the solid matrix. After hydration overnight, samples were extracted with methanol and the supernatant was collected. For NNA, the samples were analysed directly. For NNAL and iso-NNAL, a clean-up step using cation-exchange SPE was conducted; the eluent was then analysed directly by LC–MS/MS in MRM mode using ESI. Iso-NNAL isomer and NNAL isomer share a common transition, affecting their analysis. For NNAL, therefore, the analysis was performed on an isomer that is unaffected by iso-NNAL. Therefore, where iso-NNAL and NNAL were in a sample, NNAL contents were estimated, then subtracted from the combined NNAL/iso-NNAL concentration to calculate the true iso-NNAL content.

***1.21 Nitrosoacids***

Nitrosacids were measured by UK-based external laboratory. Samples were extracted with water, and an internal standard solution was added. After centrifugation, the supernatant was cleaned-up using a ChemElut diatomaceous earth cartridge, dried under nitrogen, dissolved in water, filtered and analysed by LC–MS/MS.

***1.22 N-Nitrosoalkanolamines and volatile nitrosamines***

*N*-Nitrosodiethanolamine (NDELA), *N*-nitrosodiisopropanolamine (NDiPLA), nitrosomorpholine (NMOR), *N*-nitrosodimethylamine (NDMA), nitrosomethylethylamine (NEMA), *N*-Nitrosodiisopropylamine (NDiPA), *N-*nitrosopyrrolidine (NPYR), *N*-nitrosodiethylamine (NDEA), *N*-nitrosopiperidine (NPIP), *N*-nitrosodipropylamine (NDPA) and *N*-nitrosodibutylamine (NDBA) were measured by Labstat International. Samples were extracted with distilled water using gentle heat and subjected to centrifugation. The supernatant was acidified using formic acid, combined with internal standard (deuterated analogue of the target analyte), subjected to liquid–liquid extraction, and then concentrated using dilute formic acid. The resulting sample was analysed by LC–MS/MS using a Kromasil pre-column and a hydrophilic interaction chromatography column for analyte separation and atmospheric pressure chemical ionisation (APCI) and MRM for detection**.**

***1.23 N*-Nitrosobenzylamine (NDBzA)**

Labstat International measured NDBzA. Samples were extracted by shaking with methanol and deuterium-labelled NDBzA-d12 in dichloromethane/methanol was added as internal standard. After centrifugation, the supernatant was subjected to reversed-phase SPE, evaporation and quantitative analysis by LC–MS/MS using a C18 column with APCI and MRM for detection.

***1.24 Polycyclic aromatic hydrocarbons (PAHs)***

Labstat International measured PAHs. Samples were extracted using base saponification, partitioning, SPE clean-up and concentration, followed by GC–MS. A mixture of eight deuterated PAH internal standards was added to the sample pre-extraction.

***1.25 pH***

The pH of samples was determined by Eurofins CRL using a glass electrode, pH meter and automated sampling system. Samples were extracted in water.

***1.26 Radionuclides***

Radionuclides were measured by UK-based external laboratory using standard methods for their determination in vegetable matter. Carbon-14 (^14^C) was measured by burning samples in an oxygen-rich atmosphere with a copper oxide catalyst to produce carbon dioxide, which was selectively trapped in a series of gas-bubblers containing trapping medium. Aliquots of known weight were assessed for ^14^C content by liquid scintillation counting.

For the detection of Polonium-210 (^210^Po) and Lead-210 (^210^Pb), samples were extracted with nitric acid/hydrofluoric acid and separated into two fractions for analysis of each isotope. ^210^Po was co-precipitated with iron hydroxide, dissolved and reduced in hydrochloric acid (HCl), and then auto-deposited onto a silver disk before quantification by a-spectrometry. ^210^Pb was co-precipitated with barium sulphate and then separated from barium using ethylenediaminetetraacetic acid, (EDTA) at pH 3.5. The lead sulfate precipitate was collected and stored for at least 5 days to allow Bismuth-210 (^210^Bi) to grow in. The activity of ^210^Bi was measured by gas flow proportional counting.

For the analysis of plutonium, thorium and uranium isotopes, samples were ashed at 500 °C, acid-leached, and the liquid extract split into two fractions for analysis. The first fraction was analysed for thorium isotopes (^232^Th, ^230^Th, ^228^Th). A ^229^Th internal yield tracer was added, and each sample was boiled under reflux for ~1 hour to remove carbon dioxide. After co-precipitation of the actinides with iron hydroxide, and dissolution into nitric acid, ion-exchange chromatography was used to purify and separate the thorium isotopes, which were electrodeposited onto stainless-steel discs and measured by a-spectrometry.

The second extract fraction was analysed for uranium and plutonium isotopes ^234^U/^235^U and ^238^U/^238 239,240^Pu. An internal yield tracer (U-232 or Pu-242) was added to each acid fraction and boiled under reflux for ~1 hour to remove carbon dioxide. After co-precipitation with iron, and dissolution into nitric acid, ion-exchange chromatography was used to purify and separate the plutonium and uranium isotopes, which were electrodeposited onto stainless-steel discs and measured by a-spectrometry.

For the analysis of Tritium (^3^H), samples were burnt in an oxygen-rich atmosphere with a copper oxide catalyst to produce water vapor. This vapor was selectively trapped in a series of gas bubblers containing 0.1 M nitric acid. Aliquots of this liquid were then assessed for their ^3^H content by liquid scintillation counting.

Potassium- 40 ( ^40^K), Cobalt -60 (^60^Co), Iodine-131 (^131^I), Cesium isotopes-(^134^Cs ^137^Cs), Thallium-208 (^208^Tl), Lead isotopes (^212^Pb, ^214^Pb), Bismuth isotopes (^212^Bi, ^214^Bi), (Radium-226) ^226^Ra, Actinium-228 (^228^Ac), Thorium -234 (^234^Th), Protactinium-234m (^234m^Pa) and Americium-241 (^241^Am) were measured by gamma ray spectrometry using a high-purity germanium detector coupled to an Ortec γ-ray spectroscopy system.

In all radioisotope analyses, a silicon dioxide (SiO_2_) method blank was measured at the same time as the samples. For all reported values, measurement uncertainties are quoted at 2 standard deviations (2SD) based on a total uncertainty budget.

***1.27 Sodium ions***

Eurofins CRL measured the sodium ion content using closed vessel sample digestion via nitric acid, followed by quantification using inductively coupled plasma optical emission spectrometric (ICP-OES) analysis. Sodium was measured at a wavelength/torch view of 589.592/radial.

***1.28 Sugars***

Total and reducing sugars were measured by Eurofins CRL. Samples were extracted in acetic acid, methanol and water. Reducing sugars were determined by reaction with *p*-hydroxybenzoic acid hydrazide in an alkaline media at 90 °C to form a yellow adduct. Absorbance was measured at 410 nm in a Continuous Flow Analyzer (Astoria Pacific). Total sugars were determined by using in situ hydrolysis with invertase to convert non-reducing sugars to reducing monosaccharides. Reducing sugars (original content plus converted non-reducing sugars) were then determined as described above. Calcium was used to enhance colour development for both total and reducing sugars.

***1.29 Volatile organic compounds***

1,3-Butadiene, acrylonitrile, benzene, isoprene, styrene and toluene were measured by Labstat International. Ground and homogenized samples were mixed with potassium chloride solution and d_6_-benzene internal standard in a solid-phase microextraction (SPME) vial. After headspace equilibration at room temperature, the target volatile compounds were extracted using polydimethylsiloxane-divinylbenzene fibre and analysed by GC-MS using a non-polar fused-silica capillary column for separation and full-scan mode for detection.

***1.30 Water***

Water content was measured by Eurofins CRL using Karl Fischer analysis. Samples were extracted in methanol, injected into the analysis cell, and titrated with Karl Fischer Reagent. The amount of water in the sample was calculated based on the concentration of iodine in the reagent and the amount of Karl Fischer Reagent consumed in the titration.

**Section S2. Results of method validation experiments with ONP samples:**

**Method validation approach for use of snus methods with ONPs**

The following steps were taken to compare the performance of ONPs with snus using methods pre-validated for snus.

Three test products were used in the validation process. These comprised the unflavoured zero-nicotine BAT sample, and the two competitor products. Different levels of the target analytes were added to these three validation-ONPs; the “spiked” quantities were just above the LOD to several times the LOQ. These samples were analysed alongside the study samples in the same analysis batches.

a) Extractability: For methods where digestive steps are used the laboratory visually examined samples post-digestion to confirm that both types of pouch product were equally destroyed.

b) Matrix-effects: For methods where an isotope analogue Internal Standard is added to the sample (pre- or post-extraction), all samples (both snus and ONPs) were run as a single batch, with individual replicates of samples randomised throughout the batch. The Internal Standard (IS) peak area for the pouch products was compared to that of the snus samples for which the method has been validated. Differences in IS between ONP and snus samples were flagged as a potential issue.

c) Matrix-effects: With separation-based analyses on ONPs, the retention times were examined with the spiked samples to ensure analyte retention times were not changed by the different matrices. If a study sample measurement returned a value <LOD for the main study ONP samples, the lab was asked to report whether a peak was detected at the expected position of the analyte of interest and whether it was above the noise floor.

d) Quantification: The signal peak areas for the known-quantity spiked samples were compared to the baseline noise levels to check the quantitative capability of the snus method with the ONP samples.

e) Quantification: Percentage recoveries were calculated for the spiked ONP-validation samples, taking into account the level of the analyte in the unspiked sample. Average values across the three spike-levels/products are reported.

**2.1 Summary of validation experiments:**

**2.1.1 Acrylamide:**

No issues were reported for steps (a)-(d). The average recoveries from spiked samples (step e) were 103%.

**2.1.2 Aromatic amines:**

No issues were reported for steps (a)-(d). Spiked samples gave an average recovery across the three ONPs of 93.8% for 1-aminoaphthalene, 93.9% for 2-aminoaphthalene and 85.5% for 4-aminobiphenyl.

**2.1.3 Coumarin**

No issues were reported for steps (a)-(d). Spiked samples gave an average recovery of 118.4% across the three ONPs.

**2.1.4 Ethyl carbamate**

No issues were reported for steps (a)-(d). Spiked samples gave average recoveries of 91.1%.

**2.1.5 Humectants (Glycerol and propylene glycol)**

No issues were reported for steps (a)-(d). From spiked samples, glycerol gave average recoveries of 61.1% (caused by one sample with a recovery of 4.3%; the other samples gave an average recovery of 89.5%), and propylene glycol gave average recoveries of 106.4%.

**2.1.6 Nicotine-related compounds.**

No issues were reported for steps (a)-(d). Spiked samples gave average recoveries of 115.5% for anabasine, 113.8% for anatabine, 119.4% for cotinine, 114.8% for myosmine, 161.7% for nicotine-N-oxide (one value was very elevated, thought to be due to spot contamination; the other values gave an average recovery of 124%), 120.1% for nornicotine and 115.9% with β-nicotyrine.

**2.1.7 Nitrosoalkanolamines and volatile nitrosamines**

No issues were reported for steps (a)-(d). Spiked samples gave average recoveries of 94.5% for NPIP, 102.9% for NDBA, 94.7% for NDELA, 90.1% for NDEA, 102.2% for NDiPLA, 90.7% for NDMA, 98.7% for NMOR, 100.7% for NEMA, 98.5 for NDiPA, 103.4% for NDPA and 105.9% for NPYR.

**2.1.8 N-Nitrosobenzylamine (NDBzA)**

No issues were reported for steps (a)-(d). Average recoveries of 96.1% were measured in spiked samples.

**2.1.9 Other tobacco-specific nitrosamines (NNA, NNAL, iso-NNAL):**

Standards accuracy was 86–108%, 95–113%, and 75–112% for NNA, NNAL, and iso‑NNAL, respectively, with calibration linearity (R² > 0.99) achieved across all analytes. For NNA, spike recoveries (n=1) across three products ranged from 101–118% (average 111%) at ~250 ng/g, 93–128% (average 107%) at ~625 ng/g, and 83–120% (average 100%) at ~1250 ng/g, with no analyte detected in unspiked samples above the LOD (~125 ng/g).

For NNAL, recoveries were 113–122% (average 118%), 109–113% (average 111%), and 107–114% (average 110%) at ~25, ~62.5, and ~125 ng/g, respectively, with no detection above the LOD (~12.5 ng/g). In contrast, iso‑NNAL exhibited substantially elevated recoveries due to co‑elution with NNAL (both analytes are structural isomers), with uncorrected values exceeding 200–300%. As structural isomers, NNAL and iso‑NNAL share very similar physicochemical properties, making chromatographic separation inherently challenging and prone to overlap under the current conditions. Although application of a correction reduced the bias, recoveries remained high and variable (89–224% at low, 117–227% at medium, and 110–220% at high levels), indicating persistent analytical interference and incomplete resolution between both analytes.

**2.10 Sodium:**

No issues were reported for steps (a)-(d). Average recoveries of 100.9% were obtained from spiked samples.

**2.11 Water by Karl Fisher**

No issues were reported for steps (a)-(d). Average recoveries of 99.7 % were measured with spiked samples.

**Section S3. New methods developed during the study:**

**3.1 Hydrazine:**

Standards accuracy was in the range from 88-103%. Spike at LOQ (25ng/g) had average recoveries of 103% between three products tested (n=1 replicate per product, recovery range 98%-110%). Procedural blanks had no detectable levels of hydrazine.

**3.2 Angelica lactones**

Results were reported as average of three replicates. Fortified samples were prepared by standard addition, and the recoveries were in the range of 62 – 217%. Procedural blanks were prepared in the same way with no sample included.

**3.3 Nitrosoacids**

Results were reported as average of three replicates. Fortified samples were prepared by standard addition, and the recoveries were in the range of 55 – 193 %. Procedural blanks were prepared in the same way with no sample included.

**References**

1. AOAC. Glycerol, propylene glycol, and triethylene glycol in cased cigarette cut filler and ground tobacco, gas chromatographic method 971.02. In AOAC Official Methods of Analysis 1995; Ch. 3, pp. 30.
